# Changes in the brain [NAD^+^]/[NADH] and [NADPH]/[NADP^+^] with aging and anti-aging dietary restriction

**DOI:** 10.3389/fnagi.2026.1689139

**Published:** 2026-02-04

**Authors:** Leah E. Jamerson, Tara D. Bradshaw, Patrick C. Bradshaw

**Affiliations:** 1 Department of Biomedical Sciences, James H. Quillen College of Medicine, East Tennessee State University, Johnson City, TN, United States; 2 Department of Biochemistry & Cellular and Molecular Biology, University of Tennessee, Knoxville, TN, United States

**Keywords:** aging, astrocyte, brain, dietary restriction, fasting, NAD^+^, NADPH, redox

## Abstract

Changes in brain [NADPH]/[NADP^+^] and [NAD^+^]/[NADH] may contribute to aging. Anti-aging dietary restriction (DR) and intermittent fasting (IF) alter redox states that may contribute to their longevity effects. Pyruvate/lactate and acetoacetate/beta-hydroxybutyrate are indicators of the cytoplasmic and mitochondrial [NAD^+^]/[NADH], respectively, while the malate/pyruvate and isocitrate/alpha-ketoglutarate are indicators of the cytoplasmic [NADPH]/[NADP^+^]. Using these metabolite-pair ratios as redox indicators, the C57BL/6J mouse brain showed opposite redox changes with aging to the C57BL/6N mouse brain and human brain in the cytoplasmic [NAD^+^]/[NADH] and [NADPH]/[NADP^+^]. Fasting caused universal reductive shifts in the brain cytoplasmic [NAD^+^]/[NADH] and [NADPH]/[NADP^+^] and mitochondrial [NAD^+^]/[NADH]. The reductive shift in the cytoplasmic [NAD^+^]/[NADH] with fasting was opposite to that occurring with anti-aging ketone ester supplementation or ketogenic diet, which have been shown to cause an oxidative shift of the cytoplasmic [NAD^+^]/[NADH], but a reductive shift of the cerebral cortical cytoplasmic [NADPH]/[NADP^+^]. Several pathways that influence redox metabolism and aging are discussed, including fatty acid and cholesterol synthesis, the citric acid cycle, fatty acid beta-oxidation, glutaminolysis, the malate-aspartate shuttle, the glycerol-3-phosphate shuttle, the citrate-pyruvate shuttle, and the citrate-alpha-ketoglutarate shuttle. Brain proteome, brain single-cell RNA-Seq, and brain-region-specific bulk RNA-Seq data sets of aging and DR were examined, focusing on the pathways listed above to determine how they might contribute to the redox changes. Intermittent fasting has been shown to induce cyclic metabolic switching that contributes to neuroprotection and other health benefits resulting in delayed aging, while cyclic reductive redox shifts, especially in mitochondria, may be a driver of the beneficial effects.

## 1 Introduction

According to the redox theory of aging (Go and Jones, 2017; Jones, 2015), changes in important redox couples, both extracellularly and in different subcellular compartments, contribute to aging. These redox couples include the [NADPH]/[NADP^+^], the [NAD^+^]/[NADH], the glutathione (GSH)/glutathione disulfide (GSSG), and the cysteine/cystine. Changes in the neural cell nucleocytoplasmic and mitochondrial [NADPH]/[NADP^+^] and [NAD^+^]/[NADH] will be the focus of this review. NAD(H) is a coenzyme regulating the rate of roughly 300 cellular reactions with the vast majority being regulated by the [NAD^+^]/[NADH]. Likewise, NADP(H) is a coenzyme regulating the rate of roughly 100 cellular reactions with the vast majority being regulated by the [NADPH]/[NADP^+^] (Goodman et al., 2018). So, changes in these redox ratios with aging or dietary interventions exert widespread effects on cellular metabolism (Veech et al., 2019). The review also covers the mechanisms linking the astrocyte cytoplasmic [NADPH]/[NADP^+^] and [NAD^+^]/[NADH], as these two redox couples are frequently coupled, being reduced or oxidized together. It is then highlighted how fasting causes reductive shifts in the brain cytoplasmic [NADPH]/[NADP^+^] and [NAD^+^]/[NADH] and the brain mitochondrial [NAD^+^]/[NADH], similar to what has been shown in the liver during fasting (Veech et al., 1969).

Nucleocytoplasmic [NAD^+^] increases with fasting and dietary restriction (DR) in many tissues for sirtuin deacylase activation, but nucleocytoplasmic [NADH] increases to an even greater extent, at least in the liver and in many brain regions, leading to a reduction of the redox couple. Unexpectedly, C57BL/6J and C57BL/6N mice showed opposite brain cytoplasmic redox changes with aging, which contrasted with the rat brain or mouse liver that did not show aging-related redox changes in the cytoplasmic [NADPH]/[NADP^+^] or [NAD^+^]/[NADH]. However, some evidence suggests that oxidation of the mitochondrial [NAD^+^]/[NADH] occurs in mouse neurons and perhaps other neural cell types with aging. Fasting has been shown to cause a reductive shift in the mouse brain mitochondrial [NAD^+^]/[NADH], at least in certain regions, that opposes the aging-related oxidative shift in neurons. Intermittent fasting induces brain cyclic metabolic switching (Mattson et al., 2018; Mattson, 2025) that may be driven by cyclic redox switching and lead to the prevention of the oxidation of the mitochondrial [NAD^+^]/[NADH] with aging and result in a slowing of the rate of brain aging.

## 2 The redox state of the cytoplasmic [NADPH]/[NADP^+^] and [NAD^+^]/[NADH] are linked in cells with high malic enzyme 1 (ME1) and LDH activities

In cells with at least moderate cytoplasmic malic enzyme 1 (ME1) activity, the malate/pyruvate is proportional to the cytoplasmic [NADPH]/[NADP^+^] (Jamerson and Bradshaw, 2024; Veech et al., 2019). Likewise, in cells with at least moderate cytoplasmic lactate dehydrogenase (LDH) activity, the pyruvate/lactate is proportional to the cytoplasmic [NAD^+^]/[NADH]. The presence of pyruvate in the numerator of one of the ratios and the denominator of the other inversely links these ratios together, so the two redox couples most often get reduced together. This link was reported using rat liver (Krebs and Veech, 1969) where a 48-h fast was shown to lead to the chemical reduction of both redox couples (Veech et al., 1969), which was also found following long-term DR in mice, where DR increased the hepatic cytoplasmic [NADPH]/[NADP^+^] as inferred from changes in the malate/pyruvate, and reduced the hepatic cytoplasmic [NAD^+^]/[NADH] as evidenced from changes in the pyruvate/lactate (Hagopian et al., 2009). A summary of redox changes occurring in the blood and different tissues with aging, fasting, and DR is shown in Table 1.

**TABLE 1:** Redox changes with fasting, DR, or aging.

Species	Stimulus	Tissue	Compartment	Redox ratio	Change	% of control	Method	References
Rat	48 h fast	Liver	Cytoplasm	[NADPH]/[NADP^+^]	Increase	634%	Malate/pyruvate	Veech et al., 1969
Rat	48 h fast	Liver	Cytoplasm	[NADPH]/[NADP^+^]	Increase	229%	Isocitrate/αkg	Veech et al., 1969
Rat	48 h fast	Liver	Cytoplasm	[NAD^+^]/[NADH]	Decrease	48%	Pyruvate/lactate	Veech et al., 1969
Rat	48 h fast	Liver	Mitochondria	[NAD^+^]/[NADH]	Decrease	71%	Acetoacetate/BHB	Veech et al., 1969
Mouse	Young DR	Liver	Cytoplasm	[NADPH]/[NADP^+^]	Increase	122%	Malate/pyruvate	Hagopian et al., 2009
Mouse	Aged DR	Liver	Cytoplasm	[NADPH]/[NADP^+^]	Increase	160%	Malate/pyruvate	Hagopian et al., 2009
Mouse	Young DR	Liver	Cytoplasm	[NAD^+^]/[NADH]	Decrease	90%	Pyruvate/lactate	Hagopian et al., 2003
Mouse	Aged DR	Liver	Cytoplasm	[NAD^+^]/[NADH]	Decrease	69%	Pyruvate/lactate	Hagopian et al., 2003
Mouse	Young DR	Liver	Mitochondria	[NAD^+^]/[NADH]	Decrease	64%	Acetoacetate/BHB	Hagopian et al., 2003
Mouse	Aged DR	Liver	Mitochondria	[NAD^+^]/[NADH]	Decrease	33%	Acetoacetate/BHB	Hagopian et al., 2003
Mouse	48 h fast	Liver	Mitochondria	NAD^+^/NADH	Increase	171%	Total nucleotides	Nakagawa et al., 2009
Mouse	16 h fast	Blood		[NAD^+^]/[NADH]	Decrease	31%	Pyruvate/lactate	Jeoung et al., 2012
Rat	52 h fast	Blood		[NAD^+^]/[NADH]	Increase	144%	Pyruvate/lactate	MacDonald et al., 1976
Rat	72 h fast	Blood		[NAD^+^]/[NADH]	Decrease	75%	Pyruvate/lactate	Kondoh et al., 1992
Rat	6 d fast	Muscle	Cytoplasm	[NAD^+^]/[NADH]	Increase	118%	Pyruvate/lactate	Fagan and Tischler, 1986
Rat	6 d fast	Muscle	Cytoplasm	[NAD^+^]/[NADH]	No change	100%	Pyruvate/lactate	Ibrahim et al., 2020
Rat	6 d fast	Muscle	Soleus muscle	NADPH/NADP^+^	Decrease	17%	Total nucleotides	Ibrahim et al., 2020
Rat	6 d fast	Heart		NADPH/NADP^+^	Decrease	38%	Total nucleotides	Ibrahim et al., 2020
Human	7 d fast	Blood		[NAD^+^]/[NADH]	Decrease	65%	Pyruvate/lactate	Aoki et al., 1975
Human	14 d fast	Blood		[NAD^+^]/[NADH]	Decrease	48%	Pyruvate/lactate	Aoki et al., 1975
Human	21 d fast	Blood	Arterial	[NAD^+^]/[NADH]	Decrease	50%	Acetoacetate/BHB	Aoki et al., 1975
Human	21 d fast	Blood	Venous	[NAD^+^]/[NADH]	Decrease	65%	Acetoacetate/BHB	Aoki et al., 1975
Female mice	IF	Brain cortex	Cytoplasm	[NAD^+^]/[NADH]	Decrease	65%	Pyruvate/lactate	Sorochynska et al., 2019
Female mice	3–24 h fast	Brain regions	Cytoplasm	[NAD^+^]/[NADH]	Decrease	70%–100%	Pyruvate/lactate	Shao et al., 2023
Female mice	3–24 h fast	Brain regions	Cytoplasm	[NADPH]/[NADP^+^]	Increase	100%–170%	Fumarate/pyruvate	Shao et al., 2023
C57BL/6N mice	Aging	10 brain regions	Cytoplasm	[NAD^+^]/[NADH]	Increase	200%–400%	Pyruvate/lactate	Ding et al., 2021
C57BL/6N mice	Aging	10 brain regions	Cytoplasm	[NADPH]/[NADP^+^]	Decrease	25%–40%	Pyruvate/lactate	Ding et al., 2021
C57BL/6J mice	Aging	Brain	Cytoplasm	[NAD^+^]/[NADH]	Decrease	50%–70%	Pyruvate/lactate	Dong and Brewer, 2019
C57BL/6J mice	Aging	Brain	Cytoplasm	[NADPH]/[NADP^+^]	Increase	130%–150%	Malate/pyruvate	Dong and Brewer, 2019

Since neurons and skeletal muscle express little ME1, they may be more susceptible to aging-induced cytoplasmic redox imbalance than liver or astrocytes that express higher levels (Supplementary Table 1; Saunders et al., 2018). Since astrocytes express moderate amounts of ME1 and lactate dehydrogenase subunits LDHA and LDHB (Supplementary Table 1; Saunders et al., 2018), the astrocyte [NADPH]/[NADP^+^] and [NAD^+^]/[NADH] are partially inversely linked, which may stabilize the cytoplasmic redox state against aging-related stresses. Increased redox stress correlates with loss of mitochondrial respiratory capacity with aging. Consistent with their maintained redox capacity, astrocytes from 18-months rats showed an increased mitochondrial respiratory capacity compared to those from 7-months rats (Jiang and Cadenas, 2014). Unfortunately, overexpression of cytoplasmic NADPH-generating enzymes, such as ME1, glucose-6-phosphate dehydrogenase (G6PD), or isocitrate dehydrogenase 1 (IDH1) by more than 2-fold in macrophages or white adipose tissue (Koh et al., 2004; Simmen et al., 2023), led to increased inflammation and/or adiposity from increased NADPH oxidase activity and lipid synthesis (Jamerson and Bradshaw, 2024), therefore limiting the potential utility of therapies that globally increase ME1 activity to stabilize the cytoplasmic [NADPH]/[NADP^+^] and [NAD^+^]/[NADH] with aging. Injection of a virus expressing ME1 into the vestibular nucleus of the brain induced a 2-fold induction of ME1 levels that led to improved locomotion and decreased neuroinflammation in a mitochondrial electron transport chain (ETC) complex I subunit (NDUFS4)-deficient mouse model of Leigh syndrome, consistent with neuroprotection, although survival was not affected (Del Prado et al., 2024).

## 3 During fasting and DR, the hepatic mitochondrial [NAD^+^]/[NADH] was reduced to inhibit SIRT4, while the mitochondrial [NAD^+^] increased to activate SIRT3 and SIRT5

Unlike NAD^+^ (Kory et al., 2020), NADP(H) does not appear to be transported across the inner mitochondrial membrane (IMM). So, the vast majority of mitochondrial matrix NADP^+^ is derived from phosphorylation of NAD^+^ by mitochondrial matrix NAD^+^ kinase 2 (NADK2). Thus, to better understand how the mitochondrial [NADPH]/[NADP^+^] changes with DR and fasting, it is important to understand the conditions that alter the mitochondrial matrix space [NAD^+^] and [NAD^+^]/[NADH]. The acetoacetate/beta-hydroxybutyrate in rat liver was shown to be proportional to the hepatic mitochondrial [NAD^+^]/[NADH] and it was further shown that this ratio was reduced by 2 days of fasting from a value of around 8 to a value of around 5 (Williamson et al., 1967). The same group showed that measurements of total NAD^+^ and NADH from homogenates of isolated mitochondria did not yield accurate measurements of the free levels (Krebs and Gascoyne, 1968). Another group measured the mouse liver acetoacetate/beta-hydroxybutyrate to show that the C57BL/6J mouse hepatic mitochondrial [NAD^+^]/[NADH] was reduced by DR (Hagopian et al., 2003).

Opposite to the fasting and DR-induced reductive shifts in the rodent mitochondrial (free) [NAD^+^]/[NADH], isolated mouse liver total mitochondrial NAD^+^/NADH showed an oxidative shift with fasting (Nakagawa et al., 2009). Therefore, measurements of total mitochondrial pyridine nucleotide levels are misleading when the biologically important ratio of the free nucleotides is desired. Increased hepatic mitochondrial NAD^+^ levels with fasting or DR were shown to activate mitochondrial deacylases SIRT3 and SIRT5. *In vitro* studies have confirmed that cellular SIRT1, SIRT2 [the most highly expressed sirtuin in the brain (Supplementary Table 1)], SIRT3, SIRT5, and SIRT6 are regulated by [NAD^+^] and not by the [NAD^+^]/[NADH] (Anderson et al., 2017), as non-physiological millimolar levels of NADH were required to inhibit sirtuin activity *in vitro* (Madsen et al., 2016).

In contrast to the five mammalian sirtuins listed above, yeast SIR2 (Lin et al., 2004) and mammalian mitochondrial SIRT4 were shown to be regulated by the [NAD^+^]/[NADH], not by the [NAD^+^]. Hepatic SIRT4 was shown to be inhibited by DR when the mitochondrial [NAD^+^]/[NADH] became reduced (Haigis et al., 2006), opposite to the activation of mitochondrial SIRT3 and SIRT5 by DR. Similar results were also found when using pancreatic islets or cerebellum (Haigis et al., 2006). So, a reduction in the mitochondrial [NAD^+^]/[NADH] with fasting (Veech et al., 1969; Williamson et al., 1967) or DR (Hagopian et al., 2003) occurs in several tissues. SIRT4 was also found to deacetylate and inhibit mitochondrial malonyl-coenzyme A (malonyl-CoA) decarboxylase (MLYCD) during *ad libitum* feeding to stimulate mitochondrial fatty acid synthesis, which was prevented by DR (Laurent et al., 2013). So, the mitochondrial [NADH] increased with fasting and DR more than the mitochondrial [NAD^+^], leading to SIRT4 inhibition. Kinetic studies also confirmed that SIRT4 was the first mammalian sirtuin discovered to be physiologically regulated by the [NAD^+^]/[NADH] instead of by [NAD^+^] (Pannek et al., 2017) as 30 μM NADH, a physiological concentration found in the mitochondrial matrix (Cambronne et al., 2016; Sallin et al., 2018), inhibited SIRT4 activity. The SIRT4 crystal structure further confirmed that the NAD^+^ binding site also accommodates NADH (Pannek et al., 2017). It has not yet been definitively shown whether the [NAD^+^] or the [NAD^+^]/[NADH] regulates mostly nuclear-localized SIRT7, but the very low nucleocytoplasmic [NADH] in mammalian cells suggests SIRT7 is likely regulated by [NAD^+^].

## 4 Fasting led to reductive shifts in the cytoplasmic [NAD^+^]/[NADH] and [NADPH]/[NADP^+^] in the brain cortex and hippocampus

Fasting for 3 days was shown to increase the human brain occipital lobe lactate levels by nearly two-fold (Pan et al., 2000), while a study of C57BL/6J mice showed that an overnight fast increased cortical lactate levels by 60% and increased hippocampal lactate levels by 30%, but without changing lactate levels in the thalamus, hypothalamus, or amygdala. Fasting brain pyruvate levels were not measured in that study (Kleinridders et al., 2018). In another study of C57BL/6J mice, the mice were fasted for 3, 6, 12, or 24 h, and metabolome analysis was performed on 6 brain regions and the spinal cord. Lactate levels were unchanged in most measurements with fasting, except for in the spinal cord and cortex where levels declined by roughly 10%. Fasting decreased pyruvate levels in the hippocampus, brainstem, olfactory bulb, and spinal cord, did not change pyruvate levels in the cerebral cortex and cerebellum, and increased pyruvate levels in the hypothalamus (Shao et al., 2023). Every other day feeding caused a reductive shift in the pyruvate/lactate in female mice cerebral cortex, but not in this region in male mice (Sorochynska et al., 2019). The data suggest that the pyruvate/lactate, as a measure of the cytoplasmic [NAD^+^]/[NADH], likely becomes more reduced with fasting in many mouse and human brain regions.

The cytoplasmic [NAD^+^]/[NADH] can also be determined by the tissue α-ketobutyrate/α-hydroxybutyrate, as these metabolites are also substrates of lactate dehydrogenase (Goodman et al., 2020; Sharma et al., 2021). Fasting mice for up to 24 h caused moderate increases in α-hydroxybutyrate levels in the spinal cord and all 6 brain regions measured (Shao et al., 2023), further suggesting a reductive shift of the central nervous system cytoplasmic [NAD^+^]/[NADH] by fasting. This reductive shift in the cytoplasmic [NAD^+^]/[NADH] during fasting may also occur following long-term fasting of the human brain (Owen et al., 1967). This fasting-induced reductive redox shift in the brain may be similar to that occurring with fasting in rat liver (Williamson et al., 1967) and in human plasma and skeletal muscle (Aoki et al., 1975) as measured by changes in the pyruvate/lactate. Exercise in humans was shown to increase blood lactate levels more in the fasted state than in the fed state, leading to a larger reductive shift in the pyruvate/lactate (Dohm et al., 1986).

The consequences of the fasting or DR-induced reductive shifts of the brain and liver cytoplasmic [NAD^+^]/[NADH] are currently unknown. Chronic reductive shifts in the cytoplasmic [NAD^+^]/[NADH] are associated with insulin resistance (Steiner et al., 2024) and mitochondrial disease (Goodman et al., 2020; Sharma et al., 2021). Mark Mattson’s group highlighted the importance of cyclic metabolic switching in the neural benefits of intermittent fasting (IF) (Mattson et al., 2018; Mattson, 2025). So, cycling between the normal redox state and a fasting-induced reductive redox state in the brain and liver may provide many of the benefits of DR and IF. The nucleocytoplasmic reductive shift during fasting is likely beneficial, as the [NAD^+^] levels increase to activate sirtuins and the [NADH] levels increase to an even greater extent to activate the malate-aspartate shuttle (MAS) to potentially reduce the mitochondrial matrix [NAD^+^]/[NADH], which may also play a role in neuroprotection and delayed aging. Aging chronically reduces the cytoplasmic [NAD^+^]/[NADH] in some tissues due to a decrease in cytoplasmic [NAD^+^] that decreases sirtuin activation. NAD^+^ levels have been shown to decline with aging in tissues such as adipose tissue, intestine, kidney, and skeletal muscle (McReynolds et al., 2021). Therefore, both the absolute concentrations of the oxidized and reduced forms of the redox coenzyme, their ratio, and whether the resulting changes in redox state are chronic or cyclic are all likely important factors determining the rate of tissue aging.

During fasting or DR, the brain malate/pyruvate as a measure of cytoplasmic [NADPH]/[NADP^+^] has not yet been measured. But due to the high specific activity of citric acid cycle enzyme fumarase, tissue malate and fumarate levels are in near-equilibrium, and their levels are therefore most frequently altered proportionally (Krebs, 1953). Fumarate levels were measured in different brain regions and in the spinal cord in response to fasting. Fumarate levels increased with fasting in cortex, hippocampus, hypothalamus, brainstem, olfactory bulb, and spinal cord but not in the cerebellum (Shao et al., 2023). The data, together with the data from the same study measuring pyruvate levels described above, indicate that fasting increased the fumarate/pyruvate, and so also likely increased the malate/pyruvate, indicative of the [NADPH]/[NADP^+^], in the spinal cord and several brain regions, including the hippocampus, brainstem, olfactory bulb, and potentially to a smaller extent in the cortex, while potentially decreasing in the hypothalamus (Shao et al., 2023). The reductive shift in the cytoplasmic [NADPH]/[NADP^+^] in several brain regions with fasting is consistent with the reductive shift in the cytoplasmic [NAD^+^]/[NADH] in some brain regions with fasting mentioned above and the inverse link between the pyruvate/lactate and the malate/pyruvate.

During long-term human fasting, decreased release of skeletal muscle pyruvate into the bloodstream has been hypothesized to lead to reductive shifts in the malate/pyruvate and pyruvate/lactate in the blood and other tissues throughout the body (Aoki et al., 1975; Owen et al., 1967, 1969). However, fasting for 5–10 days caused an oxidative shift in rat soleus skeletal muscle and heart total NADPH/NADP^+^ (Ibrahim et al., 2020), while rat skeletal muscle pyruvate/lactate was either unchanged (Ibrahim et al., 2020) or slightly oxidized depending upon the type of skeletal muscle, the age of the animal, or the duration of the fast (Fagan and Tischler, 1986). Fasting for 1–2 days caused an oxidative shift in rat blood (MacDonald et al., 1976) and heart pyruvate/lactate (Opie et al., 1963). However, an overnight fast in mice (Jeoung et al., 2012) or fasting for 3 days in rats led to a reductive shift in the blood pyruvate/lactate (Kondoh et al., 1992). So, during short-term fasting in mice and moderate to long-term fasting in rats and humans, there may be a transfer of reducing equivalents from the skeletal muscle to the brain and liver. This may occur through a relative decrease in the release of skeletal muscle pyruvate, compared to lactate and malate, into the bloodstream. Consistent with this, following a meal the venous blood pyruvate/lactate exiting human muscle was 12% more reduced compared to the arterial blood pyruvate/lactate entering muscle, while during long-term fasting the venous blood pyruvate/lactate exiting muscle was 28% more reduced than the arterial blood entering (Aoki et al., 1975).

## 5 Ketone ester treatment oxidized the cytoplasmic [NAD^+^]/[NADH] in the cerebral cortex and hippocampus and reduced the mitochondrial [NAD^+^]/[NADH] in the hippocampus

Fasting systemically increases ketone body levels. Treatment of the 3xTgAD Alzheimer’s disease model mice with ketone ester [(R)-3-hydroxybutyl-(R)-3-hydroxybutyrate], which is metabolized into two molecules of the ketone body beta-hydroxybutyrate, increased the cerebral cortical cytoplasmic [NAD^+^]/[NADH] by 70% (from 230 to 390) and increased the hippocampal [NAD^+^]/[NADH] by 65% (from 250 to 410), as inferred from the increased pyruvate/lactate (Pawlosky et al., 2017). This change was mostly driven by the increased pyruvate levels. Others found a ketogenic diet increased the total NAD^+^/NADH in the hippocampus, but not in the frontal cortex, of male Sprague Dawley rats (Elamin et al., 2017, 2020). Strikingly, this oxidizing shift in the [NAD^+^]/[NADH] induced by ketone ester treatment and the oxidizing shift in the total NAD^+^/NADH during a ketogenic diet were opposite to the reductive shifts that occurred in the hippocampus and cortex during fasting, as discussed above. The decreased systemic insulin levels and increased systemic glucagon levels that occur during fasting and DR may play some role, as their levels may not change to the same extent during the ketogenic diet or ketone ester consumption. Unlike mice fasted for 24–48 h that generally show a 5 to 20-fold increase in plasma beta-hydroxybutyrate levels, mice on a DR diet only showed a 40% to 2-fold increase in plasma beta-hydroxybutyrate levels (Lee J. S. et al., 2025). So, the DR-induced cytoplasmic reductive redox shift occurs independently of large changes in systemic ketone body levels.

Ketone ester treatment of the 3xTgAD Alzheimer’s mice reduced the hippocampal mitochondrial [NAD^+^]/[NADH] as indicated by the reduced acetoacetate/beta-hydroxybutyrate that changed from 9.3 to 3.4 (2.7-fold decrease), while there was no significant change in this ratio in the cerebral cortex (Pawlosky et al., 2017). Long-term fasting in humans has also been shown to induce a reductive shift in the blood acetoacetate/beta-hydroxybutyrate, indicative of the mitochondrial [NAD^+^]/[NADH], representing a summed systemic redox change. During long term human fasting, the acetoacetate/beta-hydroxybutyrate in venous blood exiting muscle and brain was slightly more reduced than the acetoacetate/beta-hydroxybutyrate in the arterial blood entering these tissues with a roughly 3-fold larger reductive shift in muscle compared to brain, while in the fasted state the acetoacetate/beta-hydroxybutyrate in blood exiting the splanchnic bed and kidney was slightly more oxidized than that entering (Aoki et al., 1975; Owen and Reichard, 1971).

## 6 Aging leads to a decrease in neuronal mitochondrial [NADH] that is restored *in vitro* by cysteine supplementation

Fluorescence lifetime measurement (FLIM) studies have shown that a decrease in mitochondrial NADH abundance occurs in cultured neurons from aged mice compared to that from young adult mice, which likely indicates an oxidation of the mitochondrial [NAD^+^]/[NADH] in aged neurons (Dong et al., 2019a). This suggests that an important mechanism of action leading to delayed brain aging by DR (Hagopian et al., 2003) or ketone ester treatment (Pawlosky et al., 2017) could be the reduction of the mitochondrial [NAD^+^]/[NADH]. A study also found that adding cysteine to isolated cultured neurons from aged mice increased the cellular cysteine/cystine and increased mitochondrial NADH levels back to the levels present in young neurons (Dong et al., 2019b). By reducing the mitochondrial [NAD^+^]/[NADH], metabolic function may be enhanced, but the mechanism through which this occurs following cysteine administration remains unexplored.

Cysteine supplementation likely increases flux through the neural cysteine catabolism enzyme mercaptopyruvate sulfurtransferase (MPST) to increase the generation of H_2_S, although MPST shows low expression in mouse neurons (Supplementary Table 1). H_2_S, when present at a high enough concentration, will inhibit mitochondrial ETC complex IV to slow NADH oxidation by ETC complex I, thereby increasing mitochondrial NADH levels to reduce the [NAD^+^]/[NADH] to potentially slow brain aging (Szabo et al., 2014). H_2_S can also donate electrons to ubiquinone (Landry et al., 2021) that may increase the mitochondrial membrane potential (ΔΨ) to create thermodynamic back-pressure to slow ETC flux to preserve mitochondrial NADH. Increases in H_2_S levels could also potentially lead to the persulfidation (sulfhydration) and activation of enzymes such as glycerol-3-phosphate dehydrogenase 1 (GPD1) or glycerol-3-phosphate dehydrogenase 2 (GPD2) that comprise the glycerol-3-phosphate shuttle (G3PS) to increase cytoplasmic NADH redox shuttling (Petrovic et al., 2025). This could lead to an oxidation of the cytoplasmic [NAD^+^]/[NADH] through the increased delivery of electrons from cytoplasmic NADH to mitochondrial ubiquinone.

Supplemental cysteine is also known to increase the rate of neuronal GSH synthesis to increase the cytoplasmic and mitochondrial GSH/GSSG, stimulating glutaredoxin-catalyzed protein cysteine deglutathionylation (Haseena et al., 2022). Although deglutathionylation of ETC complex I has been shown to increase its NADH-oxidizing activity, deglutathionylation of mitochondrial pyruvate dehydrogenase and α-ketoglutarate dehydrogenase has been shown to increase their activities to increase mitochondrial NADH generation which could potentially slow the aging-induced oxidation of the mitochondrial [NAD^+^]/[NADH]. So, during fasting and DR, redox-regulated protein deglutathionylation could potentially function together with protein persulfidation to increase the activity of pathways such as the MAS, citric acid cycle, and fatty acid beta-oxidation that reduce the mitochondrial [NAD^+^]/[NADH] to restore the mitochondrial redox state of aged neurons.

## 7 Changes in the brain cytoplasmic [NAD^+^]/[NADH] and [NADPH]/[NADP^+^] with aging are rodent strain and species specific

Through measuring the metabolome of young and aged C57BL/6J mouse brains, the lactate/pyruvate, a proxy for the cytoplasmic [NADH]/[NAD^+^], was found to increase by 34%–52% with aging, indicating a cytoplasmic reductive shift (Dong and Brewer, 2019), opposite to the oxidation of mitochondrial [NADH] that was found to occur in aged neurons (Dong et al., 2019a). The reductive shift in the C57BL/6J mouse brain lactate/pyruvate with aging was almost entirely driven by the 30%–50% decrease in pyruvate levels, while lactate and malate levels decreased by less than 10% with aging (Dong and Brewer, 2019). The reductive shift in the C57BL/6J mouse brain malate/pyruvate with aging indicated that the cytoplasmic [NADPH]/[NADP^+^] also underwent a reductive shift with aging. These reductive shifts in the cytoplasmic [NAD^+^]/[NADH] and [NADPH]/[NADP^+^] with aging corresponded very well with the reductive shifts found with aging in the total brain NAD^+^/NADH and NADPH/NADP^+^ in C57BL/6J mice (McReynolds et al., 2021).

While performing metabolome analysis of 10 brain regions of C57BL/6N mice over 4 time points across the lifespan, another group studying aging-related metabolite level changes in the brain found very different results in how the pyruvate levels changed with aging (Ding et al., 2021). Between the ages of 59 weeks (∼15 months) and 92 weeks (∼23 months), there was a 3-fold to 4-fold increase in pyruvate levels in all brain regions, while lactate levels slowly increased with aging, reaching values from insignificantly 10% higher to significantly 2-fold higher than young levels in different brain regions. The net result was a large decrease in the lactate/pyruvate in all brain regions that occurred after 15 months of age, reflecting an oxidative shift of the brain cytoplasmic [NAD^+^]/[NADH] with aging (Ding et al., 2021), opposite to the reductive shift with aging found in C57BL/6J mouse brain.

In the human brain, the total NAD^+^/NADH, as measured by magnetic resonance imaging (MRI) of NAD^+^ and NADH, was shown to become more reduced with aging (Zhu et al., 2015). But the more important human brain cytoplasmic (free) [NAD^+^]/[NADH], as measured by MRI measurements of the pyruvate/lactate, was shown to become more oxidized with aging (Uthayakumar et al., 2023). So, C57BL/6N mice appear to be a better model for the redox changes that occur with human brain aging than C57BL/6J mice. Fasting for 36 h was shown to increase cerebral blood flow (CBF) in most human brain regions, although there were a few brain regions that showed increased CBF with satiation (Tataranni et al., 1999). Lactate injection to reduce the human brain pyruvate/lactate and cytoplasmic [NAD^+^]/[NADH] also increased CBF (Mintun et al., 2004), showing that the reduction of the cytoplasmic [NAD^+^]/[NADH] is the driving force for the fasting-mediated increase in CBF (Vlassenko et al., 2006).

In the C57BL/6N mouse brain, malate levels were unchanged with aging in 6 brain regions but increased significantly from 10% to 25% in the other 4 brain regions. So, due to the greater increase in brain pyruvate levels with aging, the malate/pyruvate decreased substantially after 15 months of age, reflecting an oxidative shift of the cytoplasmic [NADPH]/[NADP^+^] with age (Ding et al., 2021), again opposite to the results obtained using C57BL/6J mice (Dong and Brewer, 2019).

Surprisingly, one group found GSH levels to increase with aging in the brainstem and cerebellum but not 3 other brain regions from C57BL/6N mice (Hussain et al., 1995). However, another group, also studying C57BL/6N mice, found that the GSH/GSSG showed a reductive shift with aging in all 10 brain regions measured between 15 months and 23 months of age (Ding et al., 2021). These results were also opposite to that of C57BL/6J mice (Wang et al., 2003). Together this data suggests that the glutathione disulfide reductase (GSR) activity remained low with aging in C57BL/6N mice, keeping the [NADPH]/[NADP^+^] and GSH/GSSG relatively unlinked. This allowed for the malate/pyruvate and [NADPH]/[NADP^+^] to be oxidized with aging while the GSH/GSSG was reduced. The increased brain GSH/GSSG from 15 to 23 months of age in C57BL/6N mice was driven largely by increased GSH levels that were accompanied by the increased levels of most other sulfur-containing compounds including cysteine, cystine, cystathionine, methionine, S-adenosylmethionine, S-adenosylhomocysteine, and ergothioneine during this time. However, GSSG levels did not change substantially with aging in most of the 10 brain regions measured but declined significantly between 15 and 23 months of age in the brainstem, thalamus, and hypothalamus, while taurine levels also declined slightly (Ding et al., 2021).

C57BL/6J male mice showed a mean lifespan roughly 8% longer than C57BL/6N males, while C57BL/6J female mice showed a mean lifespan roughly 5% longer than C57BL/6N females (Ogiso et al., 2025). Since nicotinamide nucleotide transhydrogenase (NNT), which is functional in C57BL/6N mice but not in C57BL/6J mice (Freeman et al., 2006), oxidizes mitochondrial matrix NADH to NAD^+^, the increased longevity of the C57BL/6J strain may be due in part to the reduction of the mitochondrial matrix [NAD^+^]/[NADH]. However, it is of interest to also determine if the small reductive shifts that occurred with aging in the brain cytoplasmic [NAD^+^]/[NADH] and [NADPH]/[NADP^+^] in the C57BL/6J mice contributed to their longevity compared to the larger oxidizing shifts that occurred with aging in these cytoplasmic redox couples in the C57BL/6N mice. DR induced roughly equivalent magnitudes of mean lifespan extension in C57BL/6N mice and C57BL/6J mice (Ikeno et al., 2005; Turturro et al., 1999, 2002), which is typically around 15% (Swindell, 2012).

A current paradigm in longevity research is that nucleocytoplasmic NAD^+^ levels decline with aging in one or more key tissues and restoring NAD^+^ levels leads to lifespan extension. The data presented above extends that paradigm showing that increasing the cytoplasmic [NADH] and [NADPH] more than the [NAD^+^] and [NADP^+^], at least in brain and liver, is associated with slowed tissue aging. Consistent with this, there was an oxidation of the cytoplasmic pyridine redox ratios with aging in shorter-lived C57BL/6N mice and a chemical reduction of the cytoplasmic pyridine redox ratios with aging in longer-lived C57BL/6J mice. So, DR may extend lifespan in part by preventing the aging-induced oxidation of these cytoplasmic redox ratios in C57BL/6N mice and by enhancing the aging-related reductive shifts in these cytoplasmic redox ratios in C57BL/6J mice. However, the redox ratios are likely only reduced in the fasted state and cycle back to normal following each meal of the DR diet. So, redox switching may also contribute to DR-mediated longevity.

In addition to the lack of functional NNT in C57BL/6J mice (Ronchi et al., 2013), they also lack the stop codon for the NADK2 gene, resulting in a longer, potentially unstable mitochondrial protein (Timmermans and Libert, 2018) that may further contribute to the dysfunctional mitochondrial NADPH generation and antioxidant function. There was a compensatory 7.7-fold increase in mitochondrial catalase (CAT) levels in the liver of C57BL/6J mice compared to C57BL/6N mice (Dogar et al., 2020). It is unknown if a similar compensatory response occurs in the brain that may stimulate the longevity of the C57BL/6J strain compared to C57BL/6N. But it appears that such an antioxidant response may occur in the C57BL/6J mouse brain that prevented the increase in brain pyruvate levels and the changes in the malate/pyruvate and the pyruvate/lactate that occurred in the aged C57BL/6N mouse brain (Ding et al., 2021).

Opposite to human skeletal muscle (Aoki et al., 1975), brains from adult human subjects and rats fed a normal diet showed a net uptake of lactate from the bloodstream and a net release of pyruvate. In rats these redox shifts were amplified following cerebral venous sinus stenosis (Wang et al., 2025), a type of stroke. The reductive shifts in the brain cytoplasmic pyruvate/lactate ([NAD^+^]/[NADH]) and malate/pyruvate ([NADPH]/[NADP^+^]) during the stroke may provide neuroprotection, in part through increasing CBF, similar to the reductive shifts that occur with fasting as described above.

Malate, pyruvate, and lactate were measured in the hippocampus, cortex, hypothalamus, and cerebellum of 8- and 22-months-old rats. No significant differences in the malate/pyruvate or pyruvate/lactate in any of the four brain regions were found with aging (Choi et al., 2024), suggesting there was no change in the [NADPH]/[NADP^+^] or [NAD^+^]/[NADH] with aging up to 22 months. Another study of the rat brain also found no change in the pyruvate/lactate with aging (Hoffman et al., 1985). But investigators found increased acetoacetate in the aged rat hippocampus (Choi et al., 2024) suggesting there may be an oxidizing redox shift in the acetoacetate/beta-hydroxybutyrate, reflecting the mitochondrial [NAD^+^]/[NADH] in the hippocampus with aging, consistent with the loss of NADH in mouse neuronal mitochondria with aging (Dong et al., 2019a).

There was no change in the C57BL/6J mouse hepatic mitochondrial [NAD^+^]/[NADH] with aging, as shown by measurements of the acetoacetate/beta-hydroxybutyrate (Hagopian et al., 2003), while there was also no change in the C57BL/6J mouse hepatic cytoplasmic [NAD^+^]/[NADH] or [NADPH]/[NADP^+^] with aging, as shown by measurements of the pyruvate/lactate and malate/pyruvate, respectively (Hagopian et al., 2003, 2009). So, the redox shifts that occur with aging are species and tissue-specific and may specifically affect the brain and other redox-sensitive tissues such as white adipose tissue (Jamerson and Bradshaw, 2024). Intestinal tissue of male Wistar rats fed *ad libitum* but fasted for 24 h prior to euthanasia showed a 2.5-fold increase in the oxidation state of the pyruvate/lactate between 4 and 24 months of age (Papila et al., 2024). However, it is unclear if this represented an oxidation of the intestinal cytoplasmic [NAD^+^]/[NADH] with aging or the failure of aged intestine to undergo a fasting-induced reduction of the intestinal cytoplasmic [NAD^+^]/[NADH] that may occur in young rats.

Opposite to the cytoplasmic reductive shift in C57BL/6J brain with aging driven by decreased pyruvate levels, neurons isolated from a mixed C57BL/6 × 129 mouse background showed an oxidized total NADPH/NADP^+^ with aging (Ghosh et al., 2014). The redox change in aged mouse neurons, but not aged mouse liver could in part be due to the much higher ME1 activity in liver compared to neurons that links the cytoplasmic NAD(H) and NADP(H) redox couples together, stabilizing them. But another reason may also be the much higher rate of NADH-generating mitochondrial fatty acid beta-oxidation in the liver (Derous et al., 2017) than in neurons (Schönfeld and Reiser, 2013).

**FIGURE 1:**
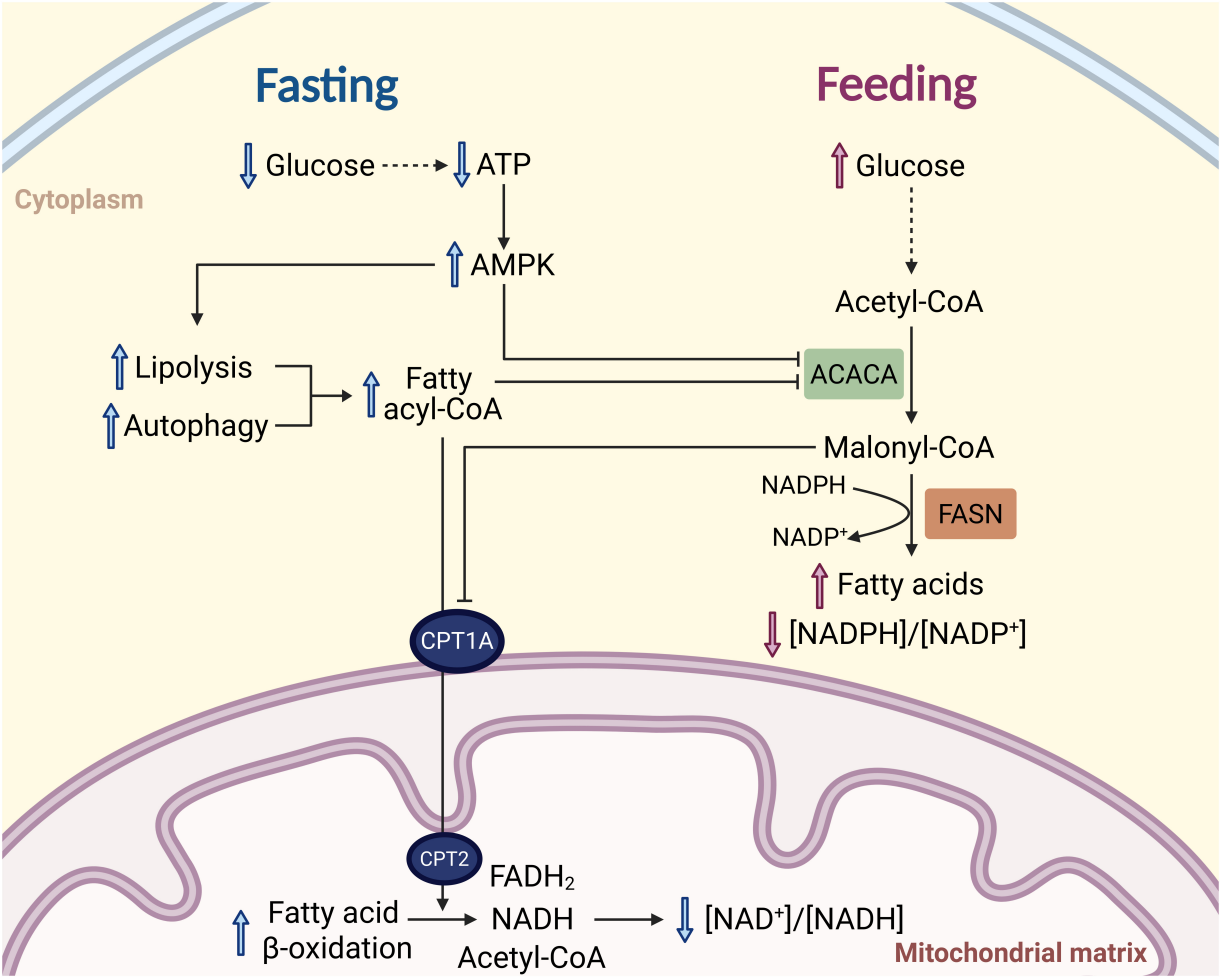
Regulation of fatty acid metabolism during fasting and feeding contributes to changes in the cytoplasmic and mitochondrial redox states. During fasting, glucose and ATP levels decline to activate AMP kinase (AMPK). AMPK phosphorylates and inhibits acetyl-CoA carboxylase alpha (ACACA) to inhibit NADPH-oxidizing fatty acid synthesis, contributing to the reduced cytoplasmic redox state during fasting. During feeding, glucose and ATP levels increase, activating ACACA to synthesize malonyl-CoA. Malonyl-CoA inhibits carnitine palmitoyltransferase 1A (CPT1A) on the outer mitochondrial membrane, which is the rate-limiting step of mitochondrial fatty acid beta-oxidation, leading to decreased NADH generation and contributing to the relatively more oxidized mitochondrial redox state when feeding.

Mitochondrial fatty acid beta-oxidation and cytoplasmic fatty acid synthesis are co-regulated to prevent futile cycling. This regulation during fasting entails adenosine monophosphate-activated protein kinase (AMPK)-mediated inhibition of acetyl-coenzyme A (acetyl-CoA) carboxylase alpha (ACACA), the rate-limiting enzyme of fatty acid synthesis, while during feeding, fatty acid synthesis metabolite and ACACA reaction product malonyl-CoA inhibits mitochondrial carnitine palmitoyltransferase 1A (CPT1A), the rate-limiting enzyme of fatty acid beta-oxidation (Figure 1). Therefore, the mitochondrial NADH generated by fatty acid beta-oxidation and the cytoplasmic NADPH preserved from inhibition of fatty acid synthesis during fasting may contribute to the reductive redox shift that occurs. In other model systems, maintained rates of fatty acid beta-oxidation with aging have been linked with longevity, while the lifespan extension in response to DR has been shown to occur through a similar mechanism as the lifespan extension that occurs by overexpression of fatty acid beta-oxidation pathway genes in *Drosophila* (Doering et al., 2023; Elmansi and Miller, 2024; Laranjeira et al., 2016; Lee et al., 2012).

## 8 Mitochondrial shuttles contribute to cellular redox homeostasis

**FIGURE 2:**
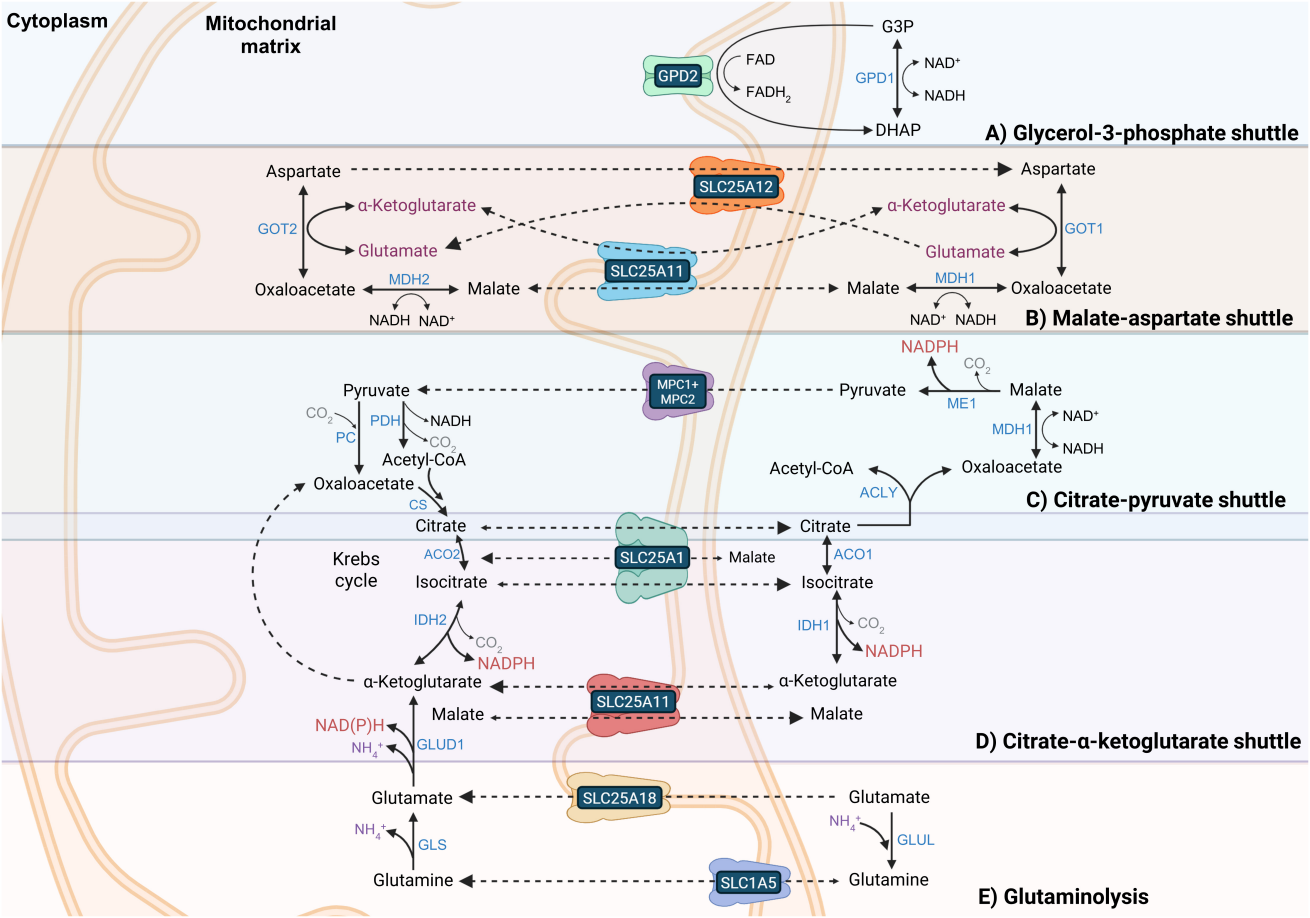
Mitochondrial shuttles play an essential role in NADPH and NADH redox metabolism. **(A)** The glycerol-3-phosphate shuttle (G3PS) shunts cytoplasmic NADH to mitochondrial intermembrane space FADH_2_ that donates electrons to ubiquinone. **(B)** The malate aspartate shuttle (MAS) shunts reducing equivalents from cytoplasmic NADH into the mitochondrial matrix to reform NADH using MDH1, GOT1, SLC25A12 or SLC25A13, SLC25A11, MDH2, and GOT2. The MAS is nearly irreversible due to the proton-motive force driving unidirectional transport of glutamate into and aspartate out of the mitochondrial matrix using either SLC25A12 or SLC25A13. **(C)** The citrate-pyruvate shuttle (CPS) not only shunts cytoplasmic NADH reducing equivalents into the mitochondrial matrix like the MAS but also synthesizes cytoplasmic acetyl-CoA and NADPH for lipid synthesis. However, since it does not return a cytoplasmic citric acid cycle intermediate to the mitochondrial matrix, it relies on pyruvate carboxylase (PC) to synthesize oxaloacetate to react with pyruvate-derived acetyl-CoA. **(D)** The citrate- α-ketoglutarate shuttle (CAS) reversibly transports NADPH reducing equivalents between the cytoplasm and mitochondrial matrix. The CAS and the CPS compete for cytoplasmic citrate. **(E)** Glutaminolysis can be used to generate mitochondrial α-ketoglutarate that feeds into the CAS, contributing to the transport of mitochondrial NADPH reducing equivalents to the cytoplasm through the reductive carboxylation of α-ketoglutarate to isocitrate by IDH2. However, mitochondria must regenerate NADPH to maintain cycle function. Transport reactions and unnamed citric acid cycle reactions are shown as dashed arrows, while other chemical reactions are shown as solid arrows. Gene symbols are shown in blue font, metabolite names are shown in black font, coenzyme names are shown in brown font, transporter names are shown in white font, and gaseous co-reactants and co-products are shown in purple font.

The G3PS (Figure 2A) and MAS (Figure 2B) are responsible for shuttling cytoplasmic NADH reducing equivalents to mitochondria for oxidation. The more reduced mitochondrial [NAD^+^]/[NADH] during fasting and DR from increased neural fatty acid oxidation (low capacity in neurons) and ketone body oxidation may slow the mitochondrial malate dehydrogenase 2 (MDH2) enzyme of the MAS, inhibiting flux through the MAS leading to the more reduced cytoplasmic [NAD^+^]/[NADH] observed in the brain of animals on the DR diet.

As part of the MAS, glutamate is transported into the mitochondrial matrix in exchange for aspartate by the SLC25A12 (Aralar) [or SLC25A13 (Citrin)] transporter. Mitochondrial glutamate is then metabolized by glutamate-oxaloacetate transaminase 2 (GOT2) to α-ketoglutarate (2-oxglutarate), which can then be exported back into the cytoplasm in exchange for malate using the SLC25A11 2-oxoglutarate carrier and transaminated back into glutamate by glutamate-oxaloacetate transaminase 1 (GOT1) to continue the MAS. The mitochondrial matrix malate is oxidized to oxaloacetate by MDH2, generating NADH, while mitochondrial oxaloacetate is transaminated into aspartate by GOT2. After the exchange of mitochondrial aspartate into the cytoplasm for glutamate, the aspartate is converted by GOT1 to oxaloacetate, which is reduced by malate dehydrogenase 1 (MDH1) back to malate with the concurrent oxidation of NADH. The cytoplasmic malate is then transported back into the mitochondrial matrix to continue shuttle function (Borst, 2020). Levels of MAS enzyme gene expression in different neural cell types of adult mice are shown in Supplementary Table 1 (Saunders et al., 2018).

The rate-limiting step of the MAS is glutamate-aspartate exchange across the IMM mediated by the SLC25A12 (Aralar) and SLC25A13 (Citrin) carriers. SLC25A12 and SLC25A13 transport activity are irreversible due to being driven by the proton-motive force across the IMM (Borst, 2020). SLC25A12 is expressed at a higher level in all adult mouse neural cell types. However, SLC25A12 and SLC25A13 expression are nearly equal in mouse oligodendrocytes (Supplementary Table 1; Ding et al., 2021; Saunders et al., 2018). MAS activity has been shown to be substantially higher in neurons than in astrocytes (McKenna et al., 2006), due in part to the over 3-fold higher expression of SLC25A12 in neurons in adult mice (Supplementary Table 1; Saunders et al., 2018). MDH2 gene expression was shown to increase with aging in one GABAergic neuron cluster in the hypothalamus (Jin et al., 2025), while MDH2 protein levels increased substantially from birth to 6 months of age but then dropped slightly from 6 to 12 months of age (Yang et al., 2008). With aging, in some neurons there was also an increased expression of GOT1 (Ximerakis et al., 2019).

A proteome study of female mice between 1 month and 22 months of age found increased abundance of all brain MAS proteins, except MDH1, with aging. SLC25A12, SLC25A13, GOT1, and GOT2 levels increased with aging in the cerebral cortex, hippocampus, and cerebellum, while MDH2 levels increased with aging in the cerebral cortex and hippocampus (Gostomska-Pampuch et al., 2021). These abundance changes are likely a compensatory response to the decreased ΔΨ that occurs with aging, decreasing SLC25A12 and SLC25A13 activity and overall flux through the MAS. SLC25A12 was shown to be persulfidated in mouse brain, but the persulfidation level did not change with DR (Bithi et al., 2021). DR was shown to increase MAS activity in the liver and kidney of aged mice (Goyary and Sharma, 2005, 2008), but the effects of DR on MAS activity in the brain have yet to be studied.

To synthesize one molecule of the 16:0 fatty acid palmitate, 14 NADPH molecules are oxidized, while to synthesize one molecule of cholesterol, 18 to 19 molecules of NADPH are oxidized (Vance and Vance, 1996). Following a meal, astrocytes have a small yet important capacity to convert glucose to fatty acids and store them as triglycerides in lipid droplets. To do this, astrocytes activate the citrate-pyruvate shuttle (CPS) (Figure 2C; Jamerson and Bradshaw, 2024), which relies upon the transport of pyruvate into the mitochondrial matrix and its metabolism to citrate via pyruvate dehydrogenase complex (PDC) and citrate synthase. Citrate is exported to the cytoplasm through the mitochondrial citrate/isocitrate carrier SLC25A1 (Jamerson and Bradshaw, 2024). ATP-citrate lyase (ACLY) catalyzes the reaction of cytoplasmic citrate with coenzyme A (CoA) to form oxaloacetate and acetyl-CoA. Malate dehydrogenase 1 (MDH1) oxidizes NADH to NAD^+^ while reducing oxaloacetate to malate, which is decarboxylated to pyruvate by ME1. The concurrent ME1-mediated reduction of NADP^+^ to NADPH provides up to half of the cytoplasmic NADPH required for fatty acid synthesis, as two NADPH molecules are oxidized for each two-carbon unit added to the growing fatty acyl chain. PPP enzymes G6PD and PGD (6-phosphogluconate dehydrogenase) generate most of the remaining NADPH required for fatty acid synthesis.

Cytoplasmic IDH1 and mitochondrial isocitrate dehydrogenase 2 (IDH2) reversibly synthesize α-ketoglutarate from isocitrate and function in the reversible mitochondrial citrate-α-ketoglutarate shuttle (CAS) that shuttles NADPH reducing equivalents between the cytoplasm and the mitochondrial matrix (Figure 2D). Citrate synthase (CS) catalyzes the reaction of acetyl-CoA with oxaloacetate to form CoA and citrate, which is then metabolized to isocitrate by the reversible enzyme aconitase 2 (ACO2). The CAS is composed of ACO2, IDH2, SLC25A11 (2-oxoglutarate carrier), IDH1, cytoplasmic aconitase 1 (ACO1), and SLC25A1 (Jamerson and Bradshaw, 2024).

In non-lipogenic tissues or in lipogenic tissues during fasted conditions when lipids are only minimally synthesized, the CAS transporters and enzymes, except ACO2 and IDH2, function in a modified version of the citric acid cycle, used to generate cytoplasmic NADPH mostly for antioxidant function (Gonthier et al., 2023). For this to occur, mitochondrial citrate is exported to the cytoplasm through SLC25A1 in exchange for malate and metabolized by cytoplasmic ACO1 to form isocitrate, which is metabolized by IDH1 to form α-ketoglutarate and NADPH. The α-ketoglutarate is then transported back into the mitochondrial matrix by SLC25A11 in exchange for malate to promote citric acid cycle function. In contrast, in lipogenic tissues under well-fed conditions, ACLY activation as a result of insulin signaling allows it to outcompete ACO1 for the metabolism of cytoplasmic citrate (Berwick et al., 2002). So, during these conditions, most citrate is metabolized into the CPS, generating both cytoplasmic acetyl-CoA and NADPH for lipid synthesis.

Proteins of the CPS and CAS are commonly post-translationally modified in response to feeding or fasting. SIRT3 was shown to deacetylate mouse mitochondrial 2-oxoglutarate carrier SLC25A11 on K73 in the brain (Dittenhafer-Reed et al., 2015), while SLC25A11 was shown to be persulfidated in mouse brain, but the persulfidation level was not altered by DR (Bithi et al., 2021). The citrate-isocitrate carrier SLC25A1 was not one of the 884 proteins found to be persulfidated in mouse brain (Bithi et al., 2021), but the *C. elegans* ortholog of SLC25A1 (K11H3.3) was shown to be persulfidated by increased H_2_S levels (Petrovic et al., 2025). The CAS may not function in all cell types, such as in human adult astrocytes, where IDH1 levels are very low, or in some cell lines (Niu et al., 2023). In the adult mouse brain, scRNA-seq studies showed that IDH2 was most highly expressed in ependymal cells, astrocytes, and microglia, with lower expression in oligodendrocytes and much lower expression in neurons (Saunders et al., 2018). ACO2 was shown to be expressed at moderately high levels in all mouse brain neural cells, with the highest levels in astrocytes and neurons, while ACO1 was expressed at moderate levels in polydendrocytes and astrocytes and at low levels in the other neural cell types (Supplementary Table 1; Saunders et al., 2018).

## 9 Increased G3PS activity may promote longevity by increasing the cytoplasmic [NAD^+^]/[NADH]

The G3PS enzyme GPD1 localizes to the cytoplasm, while the other G3PS enzyme GPD2 is attached to the intermembrane space side of the IMM (Figure 2A). The cytoplasmic GPD1L paralog of GPD1 may also contribute to G3PS function to some extent, notably in adult mouse endothelial cells, ependymal cells, fibroblast-like cells, microglia, and neurons, where GPD1L expression was shown to be higher than that of GPD1 (Supplementary Table 1; Saunders et al., 2018). However, GPD1L appears to have lower enzyme activity than GPD1, as a GPD1 null mouse showed less than 10% of the cytoplasmic glycerol-3-phosphate dehydrogenase activity of control mice in the tissues tested (Prochazka et al., 1989). GPD1 and GPD1L catalyze the reaction of glycolytic dihydroxyacetone phosphate with NADH to form glycerol-3-phosphate and NAD^+^. In tissues outside the brain, such as the liver, kidney, pancreas, and skeletal muscle, cytoplasmic GPD1 has been shown to have higher activity than mitochondrial GPD2. So, GPD2 was shown to be the rate-limiting step of the G3PS in these non-neural tissues (Eto et al., 1999) such as pancreatic beta-cells (Malaisse, 1993).

The G3PS functions in many tissues but appears to have the highest activity in skeletal muscle, brown adipose tissue, and the brain (Mráček et al., 2013). The glycerol-3-phosphate product of GPD1 can also be used for phospholipid or triacylglycerol synthesis if it is not used by GPD2 for the G3PS. GPD2 catalyzes the reverse reaction of GPD1, except a flavin adenine dinucleotide (FAD) is used as the coenzyme instead of an NADH. The FADH_2_ product of GPD2 donates its electrons to ubiquinone for ETC function. The dihydroxyacetone phosphate product of GPD2 diffuses back into the cytoplasm for use in glycolysis or for transport to peroxisomes for use in ether lipid synthesis (Oh et al., 2023). Decreased levels of ether lipids, such as plasmalogens, contribute to neuroinflammation, brain aging, and aging-related neurodegenerative diseases (Hossain et al., 2023; Jové et al., 2023).

The higher gene expression of GPD2 than GPD1 and GPD1L in mouse brain (Supplementary Table 1; Saunders et al., 2018), together with the very low GPD1 and GPD1L protein levels in mouse brain compared to skeletal muscle, liver, or kidney (Liu et al., 2021), suggest that GPD1 and GPD1L limit brain G3PS flux. Consistent with this, viral overexpression of GPD1 in the mouse brain was shown to lead to an oxidation of the cytoplasmic [NAD^+^]/[NADH] of a complex I-deficient NDUFS4^/^ mouse model, symptomized by cytoplasmic reduction of the [NAD^+^]/[NADH], which led to an extension of the mean lifespan by 44% (Liu et al., 2021). In the brain, the G3PS is likely most active in polydendrocytes and oligodendrocytes due to the moderate, roughly equal expression of both GPD1 and GPD2 in these cell types, followed by astrocytes and neurons, and then by microglia due to the lower expression levels of GPD1 and GPD1L in these cell types (Supplementary Table 1; Saunders et al., 2018). Little G3PS flux is expected in neural endothelial cells, ependymal cells, or fibroblast-like cells due to their very low expression levels of GPD1.

Expression of GPD1 and GPD2 was shown to be transcriptionally induced by a reduced cytoplasmic [NAD^+^]/[NADH] in the liver (Singh et al., 2024) to transfer the excess reducing equivalents to the mitochondria. In the human brain, an increased total NAD^+^/NADH correlated with increased ATP levels and increased membrane phospholipid turnover (Cuenoud et al., 2020), as an oxidized cytoplasmic [NAD^+^]/[NADH] likely directs glycerol-3-phosphate toward phospholipid synthesis to replace oxidized phospholipids to prevent ferroptosis instead of toward the G3PS. This may also allow the more energy-efficient MAS to shuttle cytoplasmic NADH reducing equivalents to the mitochondrial matrix for ATP synthesis when the cytoplasmic [NAD^+^]/[NADH] is more oxidized.

Glycerol-3-phosphate dehydrogenase 1 activity was shown to increase 2-fold with aging in rat brain (El-Hassan et al., 1981). However, GPD1 gene expression was shown to decline with aging in both rat oligodendrocytes and microglia, while GPD1 gene expression was increased with DR in rat oligodendrocytes (Ma et al., 2020). GPD1 gene expression increased with aging in the mouse olfactory bulb (Hahn et al., 2023) and decreased with aging in the mouse hippocampus (Schafer et al., 2015). DR increased GPD1 expression in the mouse hippocampus (Schafer et al., 2015) and led to the differential expression of GPD1 in the cerebellum (Hahn et al., 2023). So, DR could play an especially important role in enhancing the function of aged hippocampal oligodendrocytes to support neuronal and cognitive function by increasing GPD1 levels to increase G3PS activity as well as by increasing glycerol-3-phosphate levels for the synthesis of phospholipids, which contribute to the synthesis of myelin.

Mouse GPD2 expression decreased with aging in the visual cortex and motor cortex (Hahn et al., 2023). Aging increased the expression of GPD2 in some adult mouse myelinating oligodendrocytes (Jin et al., 2025). GPD2 deficiency has been shown to lead to a reduction of the nucleocytoplasmic [NAD^+^]/[NADH] that decreased the expression of mitochondrial SIRT5 desuccinylase, which increased mitochondrial protein lysine succinylation and decreased the activity of the mitochondrial fatty acid beta-oxidation pathway (Qu et al., 2024). Thus, decreased G3PS activity with brain aging may also indirectly contribute to the aging-induced oxidation of the mitochondrial [NAD^+^]/[NADH].

Unlike the MAS, the G3PS generates intermembrane space FADH_2_ instead of matrix space NADH. So, increased flux through the G3PS with aging could potentially decrease flux through the MAS and contribute to the oxidation of the mitochondrial matrix [NAD^+^]/[NADH] (Dong et al., 2019a). Consistent with this mechanism, overexpression in *C. elegans* of the glycerol-3-phosphate phosphatase PGPH-2, homologous to human candidate longevity gene PGP (Torres et al., 2021), which hydrolyzes glycerol-3-phosphate to decrease G3PS flux, extended nematode lifespan (Possik et al., 2023). Alternatively, the increased longevity could have resulted from decreased product-inhibition of the nematode homolog of cytoplasmic GPD1 by decreasing glycerol-3-phosphate levels, leading to increased flux through GPD1 to oxidize the cytoplasmic [NAD^+^]/[NADH], a known prolongevity intervention (Lautrup et al., 2024). Consistent with the latter mechanism, the H_2_S-generating compound ergothioneine was shown to stimulate GPD1 and G3PS activity to extend longevity in *C. elegans* through oxidizing the cytoplasmic [NAD^+^]/[NADH] (Petrovic et al., 2025). In that report, the increased H_2_S levels generated by transsulfuration pathway enzymes following ergothioneine treatment led to the persulfidation and increased activity of a *C. elegans* homolog of GPD1, leading to lifespan extension that was blunted when GPD1 was knocked down. The *C. elegans* homolog of GPD2 was also shown to be more persulfidated following ergothioneine treatment. In mouse brain, DR led to a 7-fold increase in GPD2 persulfidation, but no significant increase in GPD1 persulfidation (Bithi et al., 2021).

H_2_S has been shown to be essential for DR-induced longevity (Hine et al., 2015). Endogenous ergothioneine levels were shown to increase with exercise in mouse skeletal muscle, resulting in its increased binding and activation of the H_2_S-generating enzyme MPST, resulting in increased mitochondrial ETC function and increased exercise endurance (Sprenger et al., 2025), as the H_2_S generated by the metabolism of endogenous skeletal muscle ergothioneine was shown to increase mitochondrial ETC activity through the transfer of electrons from H_2_S to ubiquinone (Landry et al., 2018).

## 10 Glutamate and glutamine support mitochondrial and cytoplasmic and mitochondrial redox states

Astrocytes have been shown to have higher uptake rates of glutamine and glutamate during DR (Popov et al., 2020; Ribeiro et al., 2009). This can fuel the MAS as well as increase cytoplasmic phosphoserine aminotransferase 1 (PSAT1) enzyme activity of the serine biosynthesis pathway by mass action. Serine is the major substrate driving folate cycle flux for the generation of cytoplasmic NADPH by the enzyme ALDH1L1 (Jamerson and Bradshaw, 2024). Another way through which the increased astrocyte glutamate uptake during DR can contribute to increasing cytoplasmic NADPH generation and the [NADPH]/[NADP^+^] is through increasing the rate of glutaminolysis (Figure 2E). This entails glutamine or glutamate being transported into the mitochondrial matrix, with glutaminase (GLS) deaminating glutamine to glutamate and GLUD1 deaminating glutamate to α-ketoglutarate. While oxidizing NADPH, IDH2 can reductively carboxylate α-ketoglutarate to isocitrate, which can then be metabolized to citrate that can be exported from the mitochondrial matrix to fuel the CPS or CAS (Figures 2C,D) for cytoplasmic NADPH generation.

In the glutaminolysis-fueled cytoplasmic NADPH-generating pathway (Figure 2E), there are 4 major mechanisms available to restore the mitochondrial matrix NADPH oxidized by IDH2. These include (1) using GLUD1 for NAD(P)H generation during glutamate conversion to α-ketoglutarate instead of GOT2 that does not generate NAD(P)H, (2) increasing NNT function by increasing the levels of its substrate NADH, which occurs during times of fasting or DR as a result of an increased rate of astrocyte fatty acid and beta-hydroxybutyrate oxidation and the reduced mitochondrial [NAD^+^]/[NADH], (3) increasing the NADPH-generating flux from isocitrate through IDH2 instead of the NADH-generating flux through IDH3, or (4) increasing cataplerotic NAD(P)H-generating malic enzyme 2 (ME2) and NADPH-generating malic enzyme 3 (ME3) activities together with maintaining anaplerotic pyruvate carboxylase (PC) activity, which converts pyruvate to oxaloacetate, to balance the ME2 and ME3-mediated cataplerosis. PC was shown to be expressed at three times higher levels in adult mouse astrocytes than in neurons (Supplementary Table 1; Saunders et al., 2018), while PC activity was also shown to be more than twice as high in cultured astrocytes than in cultured neurons (Del Prado et al., 2024).

Humans also possess a brain-specific GLUD1 paralog called glutamate dehydrogenase 2 (GLUD2) that is expressed more highly in astrocytes than neurons (Nissen et al., 2017). Mouse GLUD1 was shown to be expressed at very high levels in astrocytes (Supplementary Table 1). Since the mitochondrial [NAD^+^], which is present at a concentration of ∼150–250 μM (Cambronne et al., 2016; Sallin et al., 2018), is roughly 15–100-fold higher than the mitochondrial [NADP^+^], which is present at a concentration of ∼2–10 μM, the extent of astrocytic mitochondrial NADPH generated by GLUD1 during normal *ad libitum* feeding conditions may only be moderate.

GLUD1 activity decreased with aging when measured in Albino rat brain (Rajeswari and Radha, 1984), while in Sprague-Dawley rats, GLUD1 activity in the hippocampus was shown to increase from 3 months to 12 months of age before decreasing from 12 months to 26 months of age (Bronzetti et al., 1990). The decrease in rat brain GLUD1 activity with aging may be due to oxidative damage, as GLUD1 was shown to be inactivated by carbonylation (Opii et al., 2008), nitration (Bunik et al., 2016), or glycation (Hamelin et al., 2007). A proteome study of C57BL/10J female mice found that GLUD1 levels increased with aging from 1 to 22 months in the cerebral cortex, hippocampus, and cerebellum (Gostomska-Pampuch et al., 2021) that may be a compensatory response to its oxidative inactivation with aging. However, different results were found in male BALB/c mouse brains, where GLUD1 levels did not change with aging from 3 to 26 months in the cerebral cortex, hippocampus, or striatum, but enzyme activity increased with aging in the cerebral cortex. This was due to an aging-related decrease in cortical SIRT4 expression leading to decreased GLUD1 ADP-ribosylation. Increased GLUD1 activity was further shown to stimulate astrocyte cellular senescence by increasing nucleocytoplasmic α-ketoglutarate levels and DNA and histone demethylase activities, increasing proinflammatory gene expression (Sun et al., 2023). However, in mesenchymal stem cells, the α-ketoglutarate-induced increase in histone methylation protected against cellular senescence and decreased the associated proinflammatory gene expression (Wang et al., 2020). Reviews of GLUD1 function in the aging brain have been published (Salarvandian et al., 2025; Zhou and Wang, 2025).

DR was shown to increase brain GLUD1 enzyme activity. During *ad libitum*-feeding conditions, SIRT4 was shown to ADP-ribosylate and inhibit brain GLUD1 activity which was blunted by DR (Haigis et al., 2006). The reduction of the mitochondrial [NAD^+^]/[NADH] during DR (Hagopian et al., 2003) decreased SIRT4 activity as well as its ADP-ribosylation of GLUD1, preserving GLUD1 activity (Pannek et al., 2017). In addition, knockdown of GLUD1 in cancer cells decreased the NADPH/NADP^+^ and GSH levels. Glutamate catabolism through GLUD1 was shown to increase cytoplasmic H_2_O_2_-detoxifying activity. The GLUD1 product α-ketoglutarate was shown to be metabolized in the citric acid cycle to malate, which was shown to be exported from the mitochondrial matrix and converted to fumarate that was shown to bind and activate glutathione peroxidase 1 (GPX1), which detoxifies H_2_O_2_ (Jin et al., 2015). Mouse brain GLUD1 has also been shown to be beta-hydroxybutyrylated, with beta-hydroxybutyrylation decreasing from 2 to 8 months of age (Han et al., 2024), while *C. elegans* glutamate dehydrogenase GDH-1 was shown to be persulfidated at times when H_2_S levels increased (Petrovic et al., 2025). H_2_S levels increased during DR (Hine et al., 2015), which in mouse brain led to a 5-fold increase of GLUD1 persulfidation (Bithi et al., 2021). Kinetic studies determining the effects of beta-hydroxybutyrylation and persulfidation on GLUD1 activity are needed.

Increased beta-hydroxybutyrate levels during fasting signal through protein lysine beta-hydroxybutyrylation events that alter enzyme activity (Cornuti et al., 2023; Ito et al., 2024). Beta-hydroxybutyrate-mediated inhibition of HDAC activity (Shimazu et al., 2013) may also lead to gene expression changes that facilitate a change in cellular redox state. In addition, increased ketone body levels increase flux through mitochondrial beta-hydroxybutyrate dehydrogenase 1 (BDH1) that reduces the mitochondrial [NAD^+^]/[NADH] leading to increased ETC activity (Opii et al., 2008).

## 11 The brain glutamate-glutamine cycle

Mouse astrocytes and oligodendrocytes have very high expression of cytoplasmic glutamine synthetase (GLUL) (Supplementary Table 1; Saunders et al., 2018). As part of the glutamate-glutamine cycle, astrocytes and oligodendrocytes take up the neurotransmitter glutamate released from neurons at synapses, metabolize it to glutamine using GLUL, and then export it back to neurons, where glutamine is converted back to glutamate using glutaminase (GLS). GLS was shown to be differentially expressed with mouse aging in the subventricular zone (Hahn et al., 2023), while glutaminase 2 (GLS2), expressed at a 10-fold lower level in the brain than GLS (Saunders et al., 2018), was differentially expressed with mouse aging in the olfactory bulb (Hahn et al., 2023). In *C. elegans*, glutaminase activity was shown to be increased by DR, while glutaminolysis was shown to be essential for DR-mediated longevity (Li et al., 2024).

GLUL expression increased with aging in some mouse tanycytes (Jin et al., 2025), which possess hypothalamic stem cell-like behavior (Yoo and Blackshaw, 2018), and oligodendrocytes and decreased with aging in mouse OPCs and microglia (Ximerakis et al., 2019). GLUL was differentially expressed in 2 of 15 mouse brain regions with aging (Hahn et al., 2023). GLUL protein levels increased slightly with aging in mouse hippocampus (Gostomska-Pampuch et al., 2021), which was decreased by DR (Ribeiro et al., 2009). DR also led to a 2-fold increase in GLUL persulfidation in mouse brain (Bithi et al., 2021). So, by decreasing hippocampal GLUL levels, DR may increase the flux of cytoplasmic glutamate into the mitochondrial matrix, where it is metabolized by GLUD1 or GOT2, contributing to anaplerosis and redox flexibility.

The gene expression of plasma membrane glutamate transporter SLC1A2 (EAAT2) declined with aging in mouse endothelial cells, pericytes, microglia, and neurons (Ximerakis et al., 2019), including some hypothalamic GABAergic neurons (Ximerakis et al., 2019), whereas SLC1A2 expression increased with aging in some tanycytes (Jin et al., 2025). DR increased the protein levels of SLC1A2 in rat brain (Ribeiro et al., 2009) that led to increased astrocyte glutamate uptake (Santin et al., 2011). The expression of astrocyte plasma membrane glutamate transporter SLC1A3 (EAAT1) decreased with aging in the mouse cerebellum and olfactory bulb (Hahn et al., 2023) but increased with aging in rat microglia (Ma et al., 2020). SLC1A2 and SLC1A3 have also been shown to transport cysteine, but their K_*m*_ values for cysteine transport are 5-fold and 10-fold higher, respectively, than that of SLC1A1 (EAAT3) (Zerangue and Kavanaugh, 1996). So, *in vivo* SLC1A2 and SLC1A3 may not transport much cysteine, the limiting amino acid for GSH synthesis. SLC1A1, SLC1A2, and SLC1A3 have been shown to have redox-active cysteine residues where oxidation inhibited transport (Trotti et al., 1998). Consistent with the presence of redox-active cysteines, mouse brain SLC1A2, SLC1A3, and SLC1A4 (ASCT1) were all shown to be persulfidated, although persulfidation levels did not change significantly with DR (Bithi et al., 2021). Instead of glutamate, SLC1A4 transports mostly alanine, cysteine, serine, and threonine.

## 12 Fasting does not consistently activate AMPK or decrease acetyl-CoA levels in the rodent cerebral cortex

In many tissues and brain regions during fasting, ACACA and the rate limiting enzyme of cholesterol synthesis 3-hydroxy-3-methylglutaryl-CoA reductase (HMGCR) are phosphorylated by AMPK to inhibit lipid synthesis (Muraleedharan and Dasgupta, 2022). In the brain, AMPK was shown to be activated by fasting or DR in the hypothalamus (Minokoshi et al., 2004), hippocampus (Dagon et al., 2005), and striatum (Bayliss et al., 2016). However, fasting or DR did not consistently activate AMPK in the cerebral cortex (Cantó and Auwerx, 2011), especially in female mice (Sudasinghe et al., 2025). However, in cerebral cortical astrocytes during DR, the increased autophagy and lipid droplet lipolysis may increase the levels of fatty-acyl-CoA, an inhibitor of ACACA, as an alternative mechanism to inhibit fatty acid synthesis (Goodridge, 1972), although the lack of AMPK activation would blunt lipolysis and autophagy. Likewise, the decreased systemic insulin levels during fasting have been shown to decrease the gene expression of cerebral cortical SREBF2, which encodes sterol regulatory element binding protein-2 (SREBP-2), a master transcriptional regulator of cholesterol synthesis (Suzuki et al., 2010). These findings suggest that even if cerebral cortical AMPK is not activated by fasting, lipid biosynthesis will likely decrease.

Cytoplasmic NADPH generation from the CPS relies upon cytoplasmic ACLY metabolizing CoA and mitochondrial-exported citrate for the generation of oxaloacetate and acetyl-CoA. In tissues outside the brain, fasting robustly decreased cytoplasmic acetyl-CoA levels (Eisenberg et al., 2014; Mariño et al., 2014) due to reduced insulin signaling decreasing ACLY expression (Liang et al., 2002; Shimano et al., 1999) and reduced AKT-mediated phosphorylation (Hussain et al., 2020) and stability of ACLY (Migita et al., 2008) inhibiting lipid synthesis. In contrast, DR had no effect on brain ACLY expression (Hahn et al., 2023). Consistent with this, one group found no change in brain cytoplasmic acetyl-CoA levels during fasting (Mariño et al., 2014), while another group found only a slight decrease (Son et al., 2019). However, flux through ACLY in astrocytes during DR or fasting may be product-inhibited by high levels of acetyl-CoA, especially when the next step of fatty acid synthesis, catalyzed by ACACA, is also inhibited by the high levels of fatty-acyl-CoA (Kim, 1983) present during fasting. So, the fasting-induced inhibition of lipid synthesis that occurs in the liver, white adipose tissue, and muscle appears to be blunted in the cerebral cortex due to limited AMPK activation.

In a macrophage cell line, acetylation of the mitochondrial citrate transporter SLC25A1 on K97 on the mitochondrial matrix side of the IMM was shown to stimulate its transport activity, especially under low glucose and pro-inflammatory conditions, and the acetylation event was opposed by SIRT3-mediated deacetylation (Palmieri et al., 2015). So, neural cells in the cerebral cortex that have low SIRT3 expression (Supplementary Table 1) may be able to maintain some flux through SLC25A1 and the CPS or CAS during DR for the generation of cytoplasmic NADPH. ScRNA-seq studies of adult mouse brain showed the highest SLC25A1 gene expression in oligodendrocytes and endothelial cells, followed by astrocytes. Microglia showed roughly 2-fold lower, and neurons showed roughly 4-fold lower SLC25A1 expression than astrocytes (Supplementary Table 1; Saunders et al., 2018). However, cholinergic neurons and interneurons showed roughly 30-fold higher SLC25A1 and ACLY gene expression than the average neuron (Saunders et al., 2018), to provide the cytoplasmic acetyl-CoA for acetylcholine synthesis.

## 13 Neural cell type-specific responses that drive reductive shifts in the cytoplasmic [NAD^+^]/[NADH] and [NADPH]/[NADP^+^] with fasting and DR

It is largely unknown how different tissues and cell types differentially modulate their redox state in response to diet. In liver a reduction of the nucleocytoplasmic [NAD^+^]/[NADH], such as that occurring during DR or following a high carbohydrate meal, has been shown to increase nucleocytoplasmic glycerol-3-phosphate levels (Tiwari et al., 2025) that activate the transcriptional regulator carbohydrate response element binding protein (CHREBP, encoded by the MLXIPL gene) (Singh et al., 2024). CHREBP induces hepatic lipogenic gene expression, including expression of ME1, PGD, ACLY, ACACA, and FASN (Singh et al., 2024), where ME1 and PGD contribute to an increased cytoplasmic [NADPH]/[NADP^+^], although, this increase is limited by the FASN-mediated oxidation of NADPH. In brain CHREBP is mostly expressed in microglia (Supplementary Table 1), where it has been shown to be involved in high glucose-induced proinflammatory gene expression (Yao et al., 2024). During DR microglial CHREBP may become activated by the reduced cytoplasmic [NAD^+^]/[NADH] to increase the generation of NADPH to facilitate the reductive shift in the cytoplasmic [NADPH]/[NADP^+^] to fuel antioxidant enzymes and lipid synthesis (Hagopian et al., 2009). In addition, if was shown to blunt obesity-induced cognitive dysfunction by inducing gut microbiota changes that enhanced microglial phagocytic function to decrease neuroinflammation (Mela et al., 2025).

It is unknown how DR alters the neuronal cytoplasmic [NADPH]/[NADP^+^]. The decreased fatty acid synthesis and decreased mitochondrial H_2_O_2_ generation during DR would tend to chemically reduce the redox state, while the decreased PPP flux would tend to oxidize it. The effects are likely brain region-specific (Pawlosky et al., 2017). Astrocytes fuel lipid synthesis in part by maintaining a high rate of PPP-mediated NADPH generation. Unlike other cell types where mitochondrial-derived H_2_O_2_ activated PPP flux (Talwar et al., 2023), astrocytes needed to be incubated with microglia and LPS to stimulate nitric oxide generation that activated PPP flux (Esteras et al., 2023; Iizumi et al., 2016).

## 14 Increasing flux through the MAS, citric acid cycle, or fatty acid beta-oxidation could blunt the aging-induced oxidative shift in the mitochondrial [NAD^+^]/[NADH] to slow tissue aging

The combined activities of the citric acid cycle, MAS, and fatty acid beta-oxidation that generate mitochondrial NADH appear to decline to a greater extent with aging than the activity of NADH-consuming ETC complex I in neurons, leading to an oxidized mitochondrial [NAD^+^]/[NADH] in aged neurons (Dong et al., 2019a). However, a proteome study of mouse cerebral cortex, hippocampus, and cerebellum aging did not find evidence for decreased abundance of citric acid cycle enzymes with aging (Gostomska-Pampuch et al., 2021). But, the activity of the redox-sensitive citric acid cycle enzyme ACO2 in the brain has consistently been shown to decrease with aging (Sato et al., 2022), while DR was shown to increase mouse brain ACO2 persulfidation by 2-fold (Bithi et al., 2021), which may prevent ACO2 cysteine oxidation to preserve its activity. In addition, in rat brain, the activities of succinate dehydrogenase (Kugler et al., 1988) and NAD^+^-dependent IDH3 (Patel, 1977), a rate-limiting step of the citric acid cycle (Chen and Ding, 2023), were also shown to decline with aging (Haripriya et al., 2004), which, together with the loss of ACO2 activity, may be major driving forces behind this oxidizing shift in the neuronal mitochondrial [NAD^+^]/[NADH] with aging.

A proteome study of mouse cerebral cortex, hippocampus, and cerebellum found that the abundance of enzymes of the fatty acid beta-oxidation pathway, including acetyl-CoA acetyltransferase 1 (ACAT1) and acyl-CoA dehydrogenase medium chain (ACADM), declined with aging (Gostomska-Pampuch et al., 2021), while DR led to a 4-fold increase in ACAT1 persulfidation and a decrease in ACADM persulfidation in mouse brain (Bithi et al., 2021). The gene expression of fatty acid beta-oxidation protein hydroxyacyl-CoA dehydrogenase trifunctional protein subunit beta (HADHB) also declined with aging in mouse neural endothelial cells (Ximerakis et al., 2019). Several fatty acid beta-oxidation genes were differentially expressed with aging in the mouse olfactory bulb, including ACADM, acyl-CoA dehydrogenase family member 11 (ACAD11), hydroxyacyl-CoA dehydrogenase (HADH), hydroxyacyl-CoA dehydrogenase trifunctional protein subunit alpha (HADHA), acyl-CoA synthetase medium chain family member 3 (ACSM3), and acyl-CoA synthetase medium chain family member 4 (ACSM4) (Hahn et al., 2023). Consistent with these gene expression and proteome changes, mouse brain metabolome analysis found that the ratio of C12-C18 long chain acylcarnitines to C6-C12 medium chain acylcarnitines increased with aging, suggestive of decreased long-chain fatty acid beta-oxidation in the aged brain (Borkowski et al., 2025). This decline may contribute to the aging-induced oxidation of the mitochondrial matrix [NAD^+^]/[NADH].

Neural stem cells *in vivo* have been found to rejuvenate the cells around them by secreting vesicles that increase fatty acid beta-oxidation in those nearby cells (Sun et al., 2025). Thus, these nearby neural cells are potentially being rejuvenated through a reduction of their mitochondrial [NAD^+^]/[NADH]. DR was shown to lead to the decreased persulfidation in the mouse brain of several enzymes involved in fatty acid beta-oxidation, including HADH, HADHA, HADHB, acyl-CoA dehydrogenase short chain (ACADS), enoyl-CoA hydratase 1 (ECH1), acetyl-CoA acyltransferase 2 (ACAA2), and enoyl-CoA delta isomerase 1 (ECI1) (Bithi et al., 2021).

The oxidation of the mitochondrial [NADH] in aged mouse neurons (Dong et al., 2019a) suggests that therapies to delay brain aging should be focused on therapies to increase the mitochondrial [NADH] to reduce the [NAD^+^]/[NADH], such as stimulation of the citric acid cycle (Borkum, 2023; Kurhaluk, 2024; Lin et al., 2023), fatty acid-beta-oxidation (Brandstädt et al., 2013; Liu et al., 2020), or the MAS (Easlon et al., 2008; Goyary and Sharma, 2008), ketone ester [(R)-3-hydroxybutyl-(R)-3-hydroxybutyrate] supplementation (Pawlosky et al., 2017), or slightly inhibiting the activity of mitochondrial ETC complex I (Hur et al., 2014; Stefanatos et al., 2025). Consistent with this, DR was shown to alter the expression of subventricular zone SLC25A13, one of two mitochondrial aspartate-glutamate carriers, the rate-limiting step of the MAS (Borst, 2020). Increasing the ΔΨ reduces the mitochondrial [NAD^+^]/[NADH] that may slow aging (Berry et al., 2023a,b) by both enhancing SLC25A12 and SLC25A13 activity to increase MAS activity and by creating thermodynamic back-pressure to slow ETC complex I-mediated proton pumping.

DR was also shown to increase the expression of rat brain mitochondrial ATP synthase inhibitory factor 1 (ATP5IF1/ATPIF1) (Wood et al., 2015), which inhibits IMM ATP synthase proton transport activity to increase the ΔΨ to increase the mitochondrial [NAD^+^]/[NADH] as described above. SLC25A12 and SLC25A13 activity were also shown to be stimulated by Ca^2+^ binding from the intermembrane space side of the IMM that can stimulate the MAS to reduce the mitochondrial [NAD^+^]/[NADH] to slow aging (García-Casas et al., 2018; Goyani et al., 2024). DR, other anti-aging therapies such as metformin treatment, and long-lived species compared to related short-lived species have been found to show decreased ETC complex I activity or decreased levels of ETC complex I membrane arm subunits such as NDUFV2 (Gómez et al., 2023; Miwa et al., 2014; Pamplona et al., 2021) leading to a reduction of the mitochondrial [NAD^+^]/[NADH]. So, the decreased mitochondrial ETC activity that occurs in aged brain is likely a compensatory response to the oxidized mitochondrial [NAD^+^]/[NADH] present in aged neurons and other neural cell types to attempt to restore the more reduced mitochondrial [NAD^+^]/[NADH] found in young organisms.

During the DR diet compared to glucose-oxidizing *ad libitum* fed conditions, many neural cell types will likely show a reduced mitochondrial [NAD^+^]/[NADH] from increased fatty acid beta-oxidation. So, during DR, NAD^+^-dependent PDC and citric acid cycle dehydrogenases IDH3, α-ketoglutarate dehydrogenase (OGDH), and MDH2 may be slightly inhibited, leading to decreased flux through the MAS, even though MDH2 is not the rate-limiting step of the MAS, contributing to the reduction in the brain cytoplasmic [NAD^+^]/[NADH] observed during DR. In mouse brain, DR led to 15-fold and 3-fold increases in the persulfidation of the α-ketoglutarate dehydrogenase complex subunits OGDH and DLST, respectively (Bithi et al., 2021), which could impact citric acid cycle flux and the mitochondrial [NAD^+^]/[NADH].

Therapies that stimulate transcription of mitochondrial DNA or stimulate mitochondrial ribosome activity leading to the increased abundance of mitochondrial-encoded proteins and the activity of ETC complex I (Hahn et al., 2024; Igami et al., 2024) could therefore decrease mitochondrial NADH levels, contributing to the further oxidation of the mitochondrial [NAD^+^]/[NADH] that could potentially stimulate tissue and organismal aging. However, in *Drosophila*, increased ETC complex I activity extended lifespan apparently through an oxidation of both the mitochondrial and cytoplasmic [NAD^+^]/[NADH] to increase cytoplasmic NAD^+^ levels to activate nucleocytoplasmic sirtuins to override the potential pro-aging effects of oxidation of the mitochondrial [NAD^+^]/[NADH] on longevity (Hur et al., 2014). Mitochondrial uncouplers that decrease ΔΨ, yet extend lifespan in invertebrates when administered at a low dose (Mookerjee et al., 2010), may also slow aging by oxidizing the cytoplasmic [NAD^+^]/[NADH], even though the mitochondrial [NAD^+^]/[NADH] is also oxidized. Whether this can also occur in lifespan-regulating mammalian tissues is not yet definitively known. But some evidence suggests that it is possible, as administration of the mitochondrial uncoupler 2,4-dinitrophenol was shown to extend the lifespan of mice in one study (Caldeira da Silva et al., 2008), but not in another (Miller et al., 2024). In addition, knockdown of mitochondrial NADH-generating citric acid cycle enzyme MDH2 in mice extended lifespan (Mao et al., 2025), which may have been due to an oxidizing shift in both the mitochondrial and cytoplasmic [NAD^+^]/[NADH], although the potential mechanism driving the oxidation of the cytoplasmic [NAD^+^]/[NADH] is unclear.

Mitochondrial MDH2 knockdown could have extended mouse lifespan by activating a prolongevity mitochondrial unfolded protein response (UPR*^mt^*) gene expression program that enhances the cellular redox state downstream of initial detrimental redox changes, possibly by inducing the expression of LDH or G3PS enzymes to compensate for the oxidation of the mitochondrial [NAD^+^]/[NADH]. Consistent with this, activation of the UPR*^mt^* in *C. elegans* was shown to extend lifespan without restoring the ΔΨ (Xin et al., 2022) needed for optimal glutamate-aspartate exchange and MAS flux. DR was shown to increase MDH2 persulfidation by 2-fold in mouse brain (Bithi et al., 2021) that could potentially modify MAS and citric acid cycle flux. The current knowledge of the effects of changes in brain redox state on organismal longevity suggests that cyclic reductive shifts in the neural mitochondrial [NAD^+^]/[NADH] are associated with lifespan extension. However, cyclic reductive shifts in the neural cytoplasmic [NAD^+^]/[NADH] as a result of IF, or stable oxidation of the neural cytoplasmic [NAD^+^]/[NADH] as a result of a ketogenic diet have also both been associated with anti-aging interventions. Therefore, more research is needed to confirm these initial observations and to determine the specific cell types, tissues, and brain regions where the redox changes take place.

## 15 FLIM studies using *C. elegans* showed that mid-life mitochondrial NAD(P)H can be used to determine the length of the remaining lifespan

In mammalian cells, the FLIM signal from mitochondrial NAD(P)H fluorescence was estimated to be derived 80% from NADH and 20% from NADPH (Eng et al., 1989), while the lower cytoplasmic NAD(P)H fluorescence signal is derived approximately 60% from NADH and 40% from NADPH (Blacker and Duchen, 2016). This data is consistent with measurements from HEK293 cells in culture, where the cellular total NADPH/NADH was 0.95, while the cytoplasmic total NADPH/NADH was 1.26 and the mitochondrial total NADPH/NADH was 0.63. So, the total NADPH/NADH was twice as high in the cytoplasm as in the mitochondrial matrix (Song et al., 2024), supporting the higher contribution of NADPH to the cytoplasmic FLIM signal. The differences in the individual contributions of NADPH and NADH to the FLIM signals in the different compartments could also be due in part to the fact that in the liver, mitochondrial (free) [NAD^+^]/[NADH] was found to be 7.7, roughly one hundred-fold more reduced than the hepatic cytoplasmic [NAD^+^]/[NADH], which was found to be roughly 700 (Veech et al., 2019). The mitochondrial [NADH] of 30 μM (Zhao et al., 2011) was found to be roughly 300 times higher than the cytoplasmic [NADH] of 0.1 μM (Zhang et al., 2002), while in cultured HeLa cells the mitochondrial [NADPH] of 37 μM was shown to be only roughly 12-fold larger than the cytoplasmic [NADPH] of 3 μM (Tao et al., 2017). This data partly explains the larger contribution of NADH to the mitochondrial NAD(P)H FLIM signal.

A *C. elegans* FLIM study measuring mitochondrial NAD(P)H fluorescence with aging was conducted (Morrow et al., 2024). In *C. elegans*, changes in individual mitochondrial NADP(H) fluorescence lifetime could be measured relatively easily for the first two-thirds of the lifespan in most tissues and cell types, including body wall muscle, pharyngeal muscle, hypodermis, and germline, but it was not readily possible to obtain mitochondrial NAD(P)H measurements in neurons or the intestine (Morrow et al., 2024). With aging, the autofluorescence of worm lysosome-related organelles increased throughout the body, preventing NAD(P)H measurements at advanced age in any tissue (Coburn and Gems, 2013). Increases in *C. elegans* mitochondrial NAD(P)H fluorescence lifetime, reflecting a more oxidized environment, occurred during mid-life and slightly thereafter (adult days 5 through 11), approximately coinciding with reproductive senescence, and these changes were delayed in a long-lived *eat-2* model of DR. There was moderate natural variation in the rates of aging-induced changes in mitochondrial NAD(P)H within the WT population, and importantly, worms that maintained a youthful mitochondrial NAD(P)H fluorescence lifetime profile into mid-adulthood were shown to have extended longevity compared to individual worms that showed earlier changes in mitochondrial NAD(P)H fluorescence. Therefore, these mitochondrial NAD(P)H redox changes were used to successfully predict the length of the remaining lifespan of individual nematodes (Morrow et al., 2024). This data strongly support a role for mitochondrial [NAD^+^]/[NADH] in lifespan regulation.

## 16 Increased cytoplasmic [NAD^+^]/[NADH] increased lifespan in female *Drosophila*

Enzymes that can specifically modify the [NAD^+^]/[NADH] or [NADPH]/[NADP^+^] have been identified or engineered for use as redox-modifying tools. An enzyme called LbNOX (*Lactobacillus brevis* NADH oxidase) has been shown to increase the [NAD^+^]/[NADH] (Goodman et al., 2020). An enzyme called EcSTH (*E. coli* soluble transhydrogenase) has been shown to reduce the [NAD^+^]/[NADH] (Pan et al., 2024). And an enzyme called TPNOX (triphosphopyridine nucleotide oxidase) has been shown to oxidize the [NADPH]/[NADP^+^] (Cracan et al., 2017). These enzymes can be used as tools to test the roles of different redox couples in aging. For example, exogenous expression of cytoplasmic LbNOX was shown to extend the lifespan of female, but not male, *Drosophila*. Total pyridine nucleotide levels were measured in LbNOX-expressing fly extracts, with results showing no change in female total NAD^+^/NADH and an increase in female total NADPH/NADP^+^, whereas males showed an increased total NAD^+^/NADH and total NADPH/NADP^+^ (Yadav et al., 2026).

Metabolomics analysis of the female LbNOX transgenic *Drosophila* line that showed the largest lifespan extension showed a roughly 6-fold increase in pyruvate levels, while malate levels were relatively unchanged, lactate levels were not reported, and GSH levels were decreased by roughly 8-fold (Yadav et al., 2026). The decreased malate/pyruvate and GSH levels with LbNOX expression strongly suggest a decreased cytoplasmic (free) [NADPH]/[NADP^+^], while the large increase in pyruvate also strongly suggests that the pyruvate/lactate, a proxy for the cytoplasmic (free) [NAD^+^]/[NADH], increased, which likely drove the lifespan extension in the females. This is an example in which measurements of the total pyridine nucleotide levels led to little understanding of the underlying redox change, and discussions of the malate/pyruvate and potentially pyruvate/lactate, if available, would have conveyed a better understanding of how redox metabolism was altered.

We hypothesize that low level expression of the EcSTH targeted to the mitochondrial matrix in a model organism such as *Drosophila* or *C. elegans* may be able to reduce the oxidized mitochondrial [NAD^+^]/[NADH] present in aged tissues (Dong et al., 2019a) to extend lifespan. Previous studies expressing mitochondrial-targeted EcSTH in mammalian cells in culture using a strong promoter led to reductive stress and a decreased cellular proliferation rate (Pan et al., 2024).

## 17 Future directions: Could exogenous expression of a redox-modifying enzyme to a non-native subcellular compartment in astrocytes rescue brain aging-induced redox imbalance?

**FIGURE 3:**
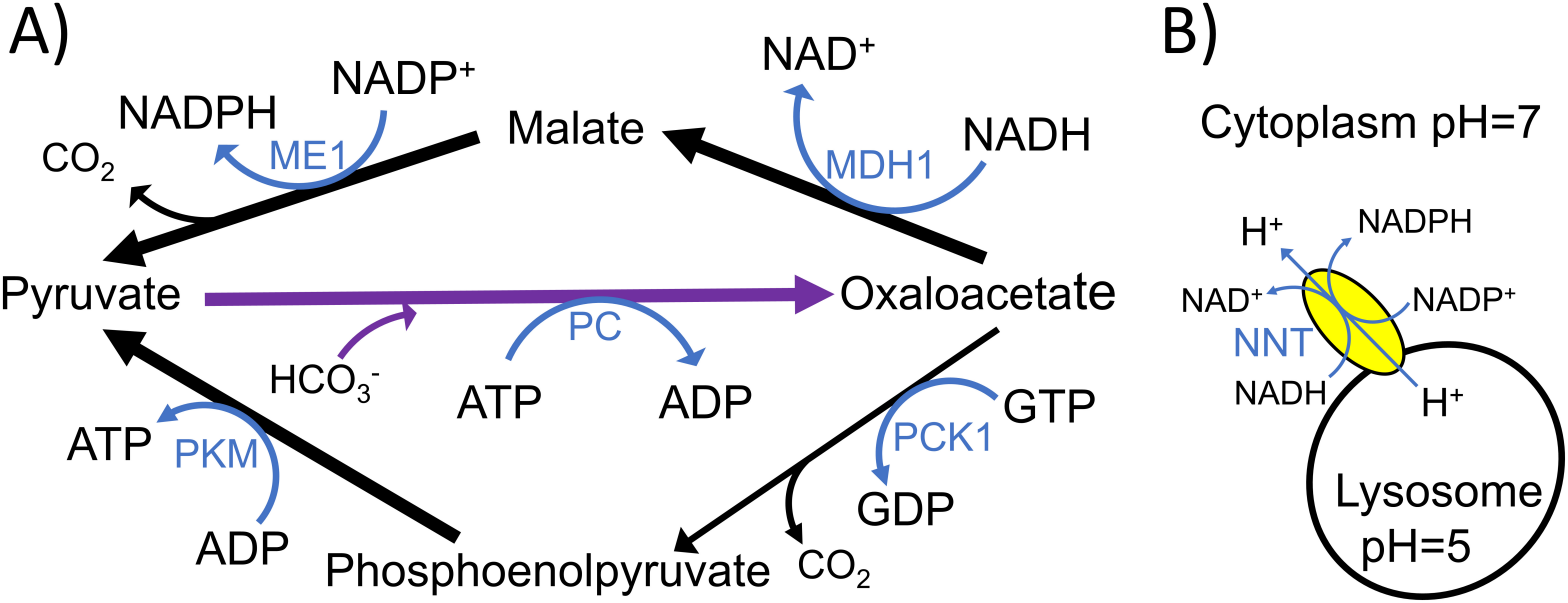
Genetic strategies to increase the cytoplasmic [NADPH]/[NADP^+^] and [NAD^+^]/[NADH]. **(A)** Exogenous expression and targeting of pyruvate carboxylase (PC) to the cytoplasm. PC is a mitochondrial matrix enzyme. Exogenous expression of a version of PC lacking the mitochondrial targeting sequence, so that it localizes to the cytoplasm, would create an energy-dissipating, redox-altering, futile cycle due to the actions of the PC, MDH1, and ME1 enzymes that effectively hydrolyze ATP to convert NADH to NADPH. The presence of the PC reaction in the cytoplasm, represented by the purple arrow in the figure, may create another futile cycle by PC functioning together with PCK1 and PKM that sums to hydrolyze GTP without altering the redox state. **(B)** Exogenous expression of a version of NNT lacking the mitochondrial targeting sequence with the addition of a secretory system signal peptide and lysosomal targeting sequence may be able to harness the lysosomal pH gradient to increase the cytoplasmic [NADPH]/[NADP^+^] and [NAD^+^]/[NADH] to blunt or reverse aging-related redox changes.

Metabolically futile, but redox-altering multi-enzyme cycles can possess transhydrogenase activity to potentially restore the cytoplasmic redox state of aged cells. In some prostate cancer cells, a portion of the PC enzyme, normally a mitochondrial matrix enzyme, became localized to the cytoplasm, which, together with MDH1 and ME1, formed a complex that catalyzed a metabolically futile cycle that hydrolyzed ATP while oxidizing NADH to NAD^+^ and reducing NADP^+^ to NADPH (Figure 3A) to improve cytoplasmic redox imbalance to bypass cellular senescence (Someya et al., 2010). So, a possible limitation of restoring the cytoplasmic redox state in aged cells may be the facilitation of tumor development from decreased cellular senescence. Cellular senescence is known to increase proinflammatory cytokine release linked to aging. So, restoring the redox state through expression of cytoplasmically-targeted PC in a tissue and cell type-specific manner in a cell type not very prone to tumor formation, but prone to cellular senescence with aging, is therefore a viable anti-aging strategy (Smith et al., 2021). Astrocytes are such a cell type that express moderate ME1 and MDH1 levels (Supplementary Table 1; Acevedo et al., 2023). Therefore, exogenous expression in aged astrocytes of a cytoplasmically targeted PC gene could possibly decrease cellular senescence and inflammation from the senescence-associated secretory phenotype (SASP) to delay brain aging.

One potential minor drawback of exogenous cytoplasmic PC expression is the activation of another metabolically futile cycle, with PC functioning together with phosphoenolpyruvate carboxykinase 1 (PCK1) and pyruvate kinase (PKM) to futilely hydrolyze GTP to GDP (Figure 3A), which could inhibit GTP-dependent processes such as endocytosis and autophagy (Santana et al., 2025). However, this would not likely be a limiting issue in astrocytes, as PCK1 was shown to be expressed at very low levels in the brain, mostly in neurons, but could be expressed in astrocytes following brain injury (Geng et al., 2021). The brain shows only limited gluconeogenesis mediated by astrocytes, possibly using the very low levels of either mitochondrial phosphoenolpyruvate carboxykinase 2 (PCK2) or cytoplasmic PCK1 (Yip et al., 2016). In the mitochondrial matrix, endogenous PC, MDH2, and either ME2 or ME3 also potentially catalyze a similar futile metabolic cycle as PC, MDH1, and ME1 in the cytoplasm. If ME2 participates in the mitochondrial cycle, the cycle would mostly result in futile ATP hydrolysis. Therefore, the expression of ME2 and ME3 evolved to be low in astrocytes where PC expression is high, and the expression of PC evolved to be low in neurons where ME3 expression is moderate (Supplementary Table 1).

Strategies that modestly increase both the cytoplasmic [NAD^+^]/[NADH] and [NADPH]/[NADP^+^] and stimulate mitochondrial NADH-generating pathways or inhibit mitochondrial NADH-oxidizing pathways in astrocytes, oligodendrocytes, and neurons will likely improve metabolic health and decrease oxidative damage to decrease cellular senescence, a driver of brain aging (Zhang et al., 2022). Astrocytes (Horn et al., 2024; Matias et al., 2022) and microglia (Ma et al., 2024; Matsudaira et al., 2023) are particularly prone to aging-induced cellular senescence, but since microglia express high levels of NADPH oxidase 2 (NOX2/CYBB), increasing NADPH levels in microglia may lead to increased H_2_O_2_ levels and proinflammatory cytokine secretion.

Most multicellular and some unicellular eukaryotes, but not some insects such as *Drosophila* species, possess a mitochondrial proton-motive force-driven transmembrane transhydrogenase homologous to human NNT (Zhang et al., 2017), while bacteria can possess a transmembrane transhydrogenase, a soluble transhydrogenase, or rarely both (Sauer et al., 2004). The IMM-localized NNT functions to synthesize NADPH using reducing equivalents from NADH in the mitochondrial matrix (Gan et al., 2024). In contrast, the soluble transhydrogenase functions in reverse to synthesize NADH using reducing equivalents from NADPH, and when overexpressed, can induce NADH-mediated reductive stress (Pan et al., 2024).

Another possible strategy to delay aging is to express a lysosome-targeted transmembrane proton-motive force-driven transhydrogenase, such as a mammalian NNT homolog, with the nucleotide binding domain facing the cytoplasm (Figure 3B). When present in the lysosomal membrane, the NNT homolog could harness the lysosomal proton gradient to synthesize cytoplasmic NADPH and NAD^+^ from NADH and NADP^+^ to potentially restore the cytoplasmic redox ratios in aged cells. It is possible that the >10-fold larger proton-gradient across the lysosomal membrane compared to the IMM or the ∼300-fold lower NADH concentration in the cytoplasm compared to the mitochondrial matrix would hinder the effectiveness of this strategy. Expression of a lysosomal-targeted NNT homolog would likely increase the cytoplasmic [NAD^+^]/[NADH] more effectively than increasing the cytoplasmic [NADPH]/[NADP^+^] due to the limiting 0.1 μM cytoplasmic [NADH] that is approximately 10–80-fold lower than the cytoplasmic [NADP^+^]. If this strategy is toxic *in vivo*, such a construct may be a useful tool to study the effects of an increased cytoplasmic [NAD^+^]/[NADH], and perhaps a slightly increased [NADPH]/[NADP^+^], in cells in culture, likely having a similar result as the exogenous expression of the LbNOX redox-modifying enzyme (Goodman et al., 2020).

## 18 Limitations to modulating the brain [NAD^+^]/[NADH] and [NADPH]/[NADP^+^] to slow aging

Although there are many reports of NAD^+^ precursor administration delaying pathology in animal models of aging-related diseases (Alegre and Pastore, 2023; Migaud et al., 2024; Sharma et al., 2023; Zhang et al., 2025), there is not an overwhelming amount of evidence showing that an aging-related decline of tissue NAD^+^ levels facilitates aging and that preventing NAD^+^ loss extends lifespan (McReynolds et al., 2020; Peluso et al., 2022), although nicotinamide mononucleotide (NMN) supplementation extended the median lifespan of female, but not male, mice by 8.5% (Kane et al., 2024). Supplementation with the NAD^+^ precursors nicotinamide, NMN, or nicotinamide riboside also improved some markers of health span in aged mice (Harrison et al., 2021; Kane et al., 2024; Mitchell et al., 2018; Zhang et al., 2016). Many of the beneficial and anti-aging effects of NAD^+^ precursor administration could be due to NAD^+^ acting as a signaling molecule to activate the mitochondrial unfolded protein response (UPR^mt^) (Mouchiroud et al., 2013).

The rate of aging may be partially controlled by cellular senescence in adipose tissue that signals for systemic inflammation (Zhang et al., 2024). DR may then slow aging by signaling through hypothalamic nuclei and brainstem nuclei that increases sympathetic nervous system signaling to induce white adipose tissue browning that delays the adipose tissue cellular senescence and inflammation (Lee A. H. et al., 2025; Sipe et al., 2017). Consistent with this, NAD^+^ levels decline with aging in some hypothalamic nuclei and the levels are restored by fasting. So, only a few tissues, blood cell types, and small brain regions may be involved in organismal lifespan determination. Consistent with this, depleting mouse skeletal muscle NAD^+^ levels by 85% did not affect muscle function, muscle aging, or organismal aging (Chubanava et al., 2025). So, the redox changes discussed throughout this review might only function to slow brain aging to delay cognitive dysfunction and neurodegenerative disease without affecting organismal aging.

Lastly, although redox coenzyme ratios change with aging in mouse brain, they remained unaltered with aging in mouse liver. Therefore, a study showed a high level of preservation of global metabolic flux with aging in C57BL/6J mice, although plasma and tissue metabolite levels varied moderately (Jankowski et al., 2025). Similar studies are needed to determine how fasting and DR alter metabolic flux throughout central metabolism. Measurements of metabolic flux in the brain are also needed with aging, fasting, and DR.

## 19 Conclusion

During mouse brain aging, the cytoplasmic [NADPH]/[NADP^+^] changed relatively independently of the GSH/GSSG. So, under *ad libitum*-fed conditions, changes in brain cytoplasmic [NADPH]/[NADP^+^] were shown to have little influence on the GSH/GSSG. Due to the lack of studies using metabolite-pair measurements of the cytoplasmic and mitochondrial (free) [NADPH]/[NADP^+^] and [NAD^+^]/[NADH] in the brain, little progress has been made on how these ratios change with aging or during anti-aging therapies such as DR. Reporting of total pyridine nucleotide levels after tissue lysis is often performed, but can yield data of little biological relevance, especially when working with isolated mitochondrial extracts. Measurements of total NADPH/NADP^+^ during fasting did not correlate to changes in the malate/pyruvate indicative of the cytoplasmic (free) [NADPH]/[NADP^+^] (Veech et al., 1969). Measurements of metabolite-pairs to determine the redox state appear to be most important for studies determining the effects of fasting or DR on the mitochondrial [NAD^+^]/[NADH], where measurements of mitochondrial total NAD^+^/NADH following fasting showed either no change or an oxidizing redox shift, opposite to the reducing redox shift indicated by measurements of the tissue acetoacetate/beta-hydroxybutyrate indicative of the mitochondrial (free) [NAD^+^]/[NADH] (Hagopian et al., 2003; Haigis et al., 2006; Nakagawa et al., 2009; Veech et al., 1969; Yang et al., 2007).

**FIGURE 4:**
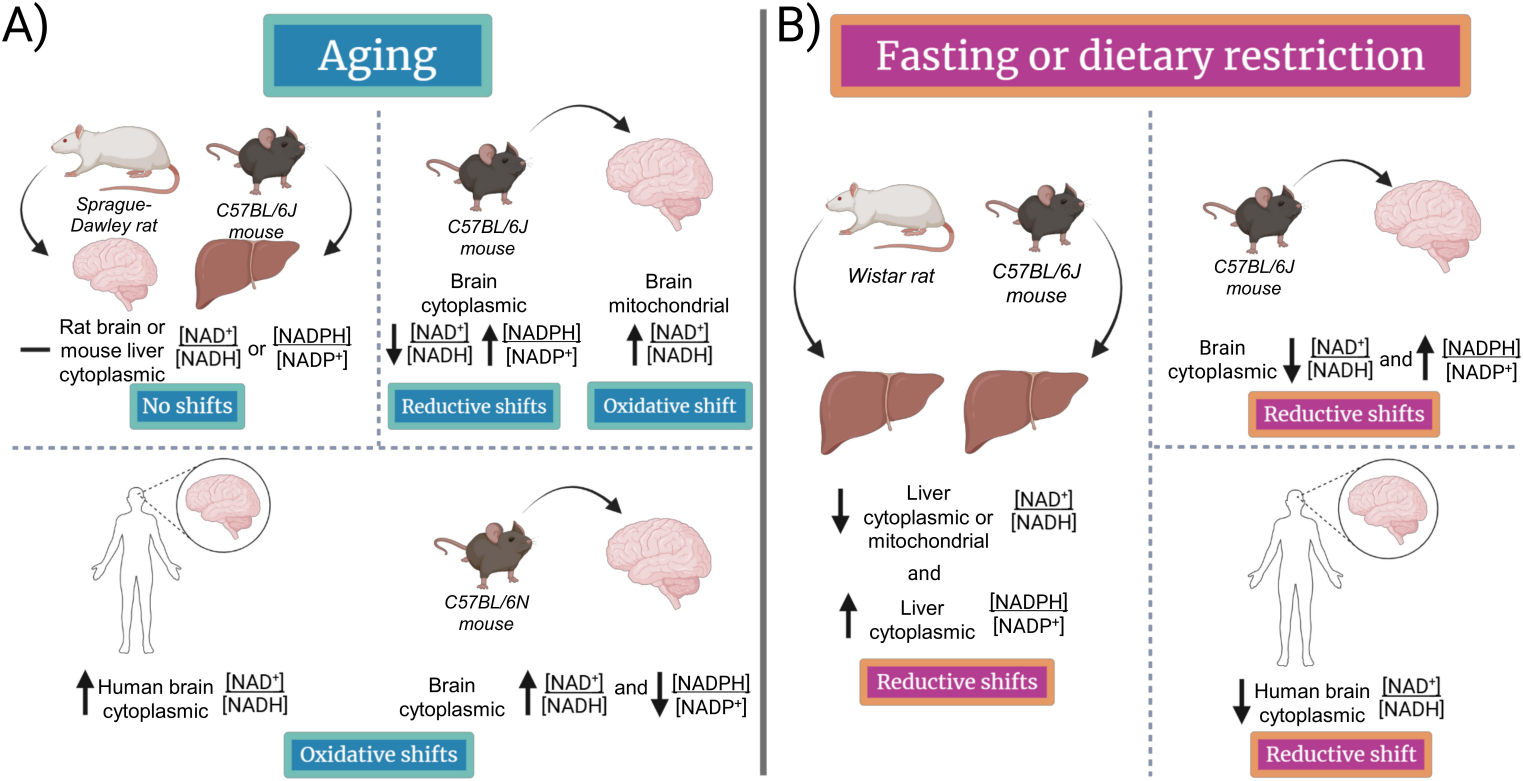
Changes in the cytoplasmic [NAD^+^]/[NADH] and [NADPH]/[NADP^+^] and mitochondrial [NAD^+^]/[NADH] with aging and fasting or dietary restriction. **(A)** In Sprague-Dawley rat brain or C57BL/6J mouse liver there were no cytoplasmic pyridine nucleotide redox changes with aging, while the C57BL/6J mouse brain showed a reductive redox shift with aging in the cytoplasmic pyridine nucleotide redox couples. In contrast, the human brain and C57BL/6N mouse brain showed an oxidative redox shift in the cytoplasmic pyridine nucleotide redox couples with aging. **(B)** With fasting or DR there were consistent reductive shifts in the cytoplasmic pyridine nucleotide redox couples and the mitochondrial [NAD^+^]/[NADH] in Wistar rat and C57BL/6J mouse liver, while fasting also induced a reductive shift in C57BL/6J mouse brain cytoplasmic pyridine nucleotide redox couples and the human brain cytoplasmic [NAD^+^]/[NADH].

Using measurements of the pyruvate/lactate, it was found that the brain cytoplasmic [NAD^+^]/[NADH] underwent a reductive redox shift in C57BL/6J mice with aging but an oxidizing redox shift in C57BL/6N mice and humans with aging. Likewise, from measurements of the brain malate/pyruvate, the cytoplasmic [NADPH]/[NADP^+^] was shown to undergo a reductive redox shift in C57BL/6J mice with aging but an oxidizing redox shift in C57/BL6N mice with aging. In each strain of mice, the redox state of the GSH/GSSG changed in the opposite direction with aging as that of the [NADPH]/[NADP^+^]. Oxidation of the brain mitochondrial [NAD^+^]/[NADH] may universally occur with organismal aging, while DR causes cyclic reductive shifts in the cytoplasmic and mitochondrial pyridine nucleotide redox ratios, as summarized in Figure 4. Studies measuring the acetoacetate/beta-hydroxybutyrate in different brain regions with aging and DR are needed to determine the extent to which DR leads to a reduction of the brain mitochondrial [NAD^+^]/[NADH], possibly driving neuroprotection and slowing the rate of brain aging.
