# Supplementary material for: Changes in the brain [NAD+]/[NADH] and [NADPH]/[NADP+] with aging and anti-aging dietary restriction
Source: Front Aging Neurosci. 2026 Feb 4;18:1689139. doi: 10.3389/fnagi.2026.1689139 (PMC12913425; doi:10.3389/fnagi.2026.1689139)
Supplement: Supplementary file 1 [file Data_Sheet_1.pdf]

Table S1. Median cell type-specific expression levels of genes influencing redox state and energy metabolism in adult mouse brain (transcripts per 100,000 in cluster).

Gene	Astrocytes	Endothelial cells	Ependymal cells	Fibroblast-like cells	Microglia	Neurons	Oligoden-drocytes	Polyden-drocytes
NADK	7.5	6.2	3.9	4.2	5.7	3.3	3.3	4.1
NADK2	6.3	1.2	6.0	1.3	1.4	0.8	1.9	1.4
G6PD	3.5	1.6	1.0	2.4	4.2	2.5	3.2	2.8
PGD	3.3	2.5	3.0	10.0	5.5	2.4	2.5	4.0
ME1	9.0	0.8	1.0	1.3	1.2	3.0	2.5	5.0
IDH1	12.0	3.0	4.2	4.2	4.5	2.4	8.0	25.0
ALDH1L1	34.0	0.7	1.6	1.7	1.3	0.4	1.0	0.4
ME2	1.4	2.4	9.4	2.7	1.9	2.2	4.4	5.3
ME3	1.0	1.1	1.1	1.4	1.4	4.8	0.5	2.7
IDH2	11.0	3.4	31.0	8.5	14.0	1.8	3.1	4.2
NNT	2.7	2.0	1.5	4.8	4.2	1.5	0.9	1.7
GLUD1	82.0	14.3	14.5	12.3	14.1	11.5	12.1	15.3
GSR	3.2	4.8	11.5	2.8	3.2	3.0	3.3	3.8
CBS	12.9	0.2	5.7	0.7	0.6	0.1	0.8	0.3
MPST	3.9	0.5	4.0	1.4	0.9	0.6	3.3	1.6
CTH	4.3	0.1	0.2	0.3	0.2	0.1	0.1	0.2
GLUL	325	170	23	165	95	15	340	32
GLS	3.3	7.3	2.4	4.8	9.1	23.0	3.0	6.0
GLS2	0.2	0.2	0.0	0.7	0.7	2.2	0.3	0.3
SLC1A1	0.7	14.9	0.5	1.3	1.8	7.2	0.8	28.0
SLC1A2	650	27.0	17.6	33.5	41.0	37.0	74.0	58.0
SLC1A3	550	8.8	44.5	72.0	24.3	4.6	22.1	17.0
SLC1A4	19.1	0.5	9.9	1.4	1.7	3.0	0.8	3.9
SIRT1	1.2	1.7	2.0	2.0	1.2	1.5	1.3	1.8
SIRT2	21.1	13.1	21.8	11.3	13.8	7.3	96.0	310.0
SIRT3	3.5	1.3	5.8	2.2	1.6	4.0	2.1	1.8
SIRT4	1.4	0.8	0.7	1.1	1.0	1.0	0.9	1.1
SIRT5	1.5	0.9	1.2	0.9	0.8	1.1	0.4	0.8
SIRT6	0.6	0.6	0.7	0.5	0.7	0.8	0.5	1.2
SIRT7	3.3	4.3	2.7	2.5	5.9	2.5	3.1	3.8
PSAT1	17.0	1.3	13.0	1.6	3.8	1.5	46.0	20.5
SLC25A1	6.5	8.0	5.0	5.0	3.5	1.6	8.0	6.5
SLC25A11	9.5	6.5	8.0	7.1	11.4	10.6	5.4	6.9
ACLY	7.0	6.2	3.8	4.5	9.0	7.0	6.5	9.5
ACACA	3.5	0.9	1.2	1.8	1.8	2.5	2.7	2.0
ACACB	1.0	2.6	1.6	0.7	0.4	0.1	0.1	0.1
MLYCD	3.4	2.8	1.9	2.3	2.6	2.7	2.3	3.2
FASN	24.0	1.6	6.0	6.0	4.0	7.5	11.0	13.5
HMGCR	12.5	2.3	2.0	2.8	3.9	7.0	4.5	12.5
CPT1A	10.4	11.3	3.3	6.1	2.8	0.2	1.4	2.8
CPT2	3.3	2.8	1.7	1.7	1.4	0.2	1.4	1.7
ACO1	4.5	2.4	1.6	2.7	1.8	1.5	2.1	5.9
MDH1	39.5	17.2	32.5	27.5	26.0	77.0	18.8	26.3

MDH2	29.0	21.8	24.8	22.4	16.4	31.0	18.6	23.9
GOT1	4.4	1.6	4.6	3.8	7.8	35.0	4.3	3.6
GOT2	2.3	4.3	2.7	4.7	4.2	13.4	4.8	6.1
SLC25A12	4.0	3.3	5.2	3.9	3.6	13.8	4.2	4.3
SLC25A13	0.3	0.2	1.3	0.3	0.7	0.3	3.8	1.1
GPD1	3.8	0.4	0.7	0.7	1.7	3.5	6.2	7.6
GPD1L	2.4	2.1	1.4	2.9	2.9	4.8	1.1	2.6
GPD2	31.0	43.0	3.4	11.9	7.4	10.7	7.8	6.9
PGP	18.2	7.2	3.7	7.7	7.4	10.2	20.8	21.6
PC	7.8	1.8	2.7	4.1	1.4	2.6	3.2	1.7
CS	31.6	8.1	7.6	8.5	7.3	14.9	8.7	9.1
ACO2	46.0	16.0	18.5	22.2	17.8	37.6	21.7	34.5
IDH3A	12.2	4.3	17.3	4.7	6.7	19.6	7.1	11.6
IDH3B	21.2	12.7	12.3	13.4	12.2	22.5	8.0	11.4
IDH3G	13.4	9.2	14.1	11.8	12.4	11.1	7.4	7.3
GPX1	6.7	56.0	16.0	27.0	42.0	7.2	6.5	11.5
ATP5IF1	21.8	27.3	69.0	37.6	47.5	46.0	22.3	40.3
GAPDH	1.4	0.5	0.6	0.5	0.7	1.3	0.7	0.6
ENO1	18.8	8.6	9.8	5.3	6.3	7.2	3.9	11.7
PKM	31.7	32.8	40.9	20.4	23.9	55.0	15.8	26.8
PCK2	1.7	2.8	1.5	3.7	3.7	2.7	0.3	1.3
LDHA	15.5	29.2	7.4	16.0	13.2	19.8	3.7	8.5
LDHB	132.0	7.2	28.0	24.0	39.0	46.0	19.5	22.2
BDH1	11.0	0.4	7.3	2.2	0.7	3.6	2.6	10.3
OGDH	10.7	10.4	12.2	10.6	9.9	19.8	8.7	8.2
DLST	5.9	3.4	5.8	4.3	4.9	6.3	3.8	4.7
ACAA2	11.8	9.2	7.5	8.2	2.9	0.5	1.0	0.5
ACADL	29.0	24.0	9.2	14.3	7.2	1.3	12.3	8.1
ACADM	12.3	11.8	17.1	11.7	7.4	2.3	5.7	7.4
ACADS	2.9	1.7	0.8	1.7	4.8	0.2	0.6	1.2
ACADVL	12.7	9.2	15.6	9.2	5.9	2.3	6.7	5.8
ACAD11	3.3	1.4	2.8	1.1	0.9	0.8	1.3	1.3
ACAT1	15.8	9.2	17.4	16.5	10.8	12.9	10.7	17.5
ACSM3	0.1	0.05	0.1	0.1	0.1	0.1	0.1	0.1
ACSM4	0.0	0.0	0.0	0.0	0.0	0.01	0.0	0.0
HADH	9.9	9.2	5.4	7.8	2.7	0.4	9.8	7.2
HADHA	17.0	11.9	8.8	10.6	9.2	3.9	13.7	17.1
HADHB	15.6	5.4	4.5	5.1	3.6	1.8	5.6	8.1
ECHS1	18.5	7.4	15.5	8.7	7.6	3.9	13.6	8.5
ECH1	16.6	22.2	22.8	18.1	9.6	2.3	15.8	10.1
ECI1	26.9	11.8	14.2	8.3	5.8	1.1	9.5	8.4
SREBF1	27.0	3.2	23.0	8.0	3.2	1.2	7.0	6.5
SREBF2	11.0	3.3	8.0	3.0	3.3	8.0	5.3	5.4
MLXIPL	0.4	0.1	0.4	0.3	7.1	0.05	0.05	0.1
CYBB	0.05	0.1	0	0.1	17.5	0.05	0.05	0.1

Data taken from http://dropviz.org (Saunders et. al, 2018).
